# High-Productivity Single-Pass Electrochemical Birch Reduction of Naphthalenes in a Continuous Flow Electrochemical Taylor Vortex Reactor

**DOI:** 10.1021/acs.oprd.2c00108

**Published:** 2022-08-24

**Authors:** Darren S. Lee, Ashley Love, Zakaria Mansouri, Toby H. Waldron Clarke, David C. Harrowven, Richard Jefferson-Loveday, Stephen J. Pickering, Martyn Poliakoff, Michael W. George

**Affiliations:** † School of Chemistry, University of Nottingham, University Park, Nottingham NG7 2RD, U.K.; ‡ Department of Mechanical and Manufacturing Engineering, University of Nottingham, University Park, Nottingham NG7 2RD, U.K.; § School of Chemistry, University of Southampton, Highfield, Southampton SO17 1BJ, U.K.

**Keywords:** Birch reduction, electroreduction, electrochemistry, dearomatization, continuous flow, Taylor vortex reactor

## Abstract

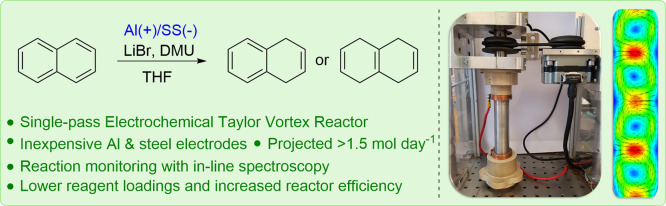

We report the development of a *single-pass* electrochemical Birch reduction carried out in a small footprint electrochemical Taylor vortex reactor with projected productivities of >80 g day^–1^ (based on 32.2 mmol h^–1^), using a modified version of our previously reported reactor [*Org. Process Res. Dev.***2021**, *25*, 7, 1619–1627], consisting of a static outer electrode and a rapidly rotating cylindrical inner electrode. In this study, we used an aluminum tube as the sacrificial outer electrode and stainless steel as the rotating inner electrode. We have established the viability of using a sacrificial aluminum anode for the electrochemical reduction of naphthalene, and by varying the current, we can switch between high selectivity (>90%) for either the single ring reduction or double ring reduction with >80 g day^–1^ projected productivity for either product. The concentration of LiBr in solution changes the fluid dynamics of the reaction mixture investigated by computational fluid dynamics, and this affects equilibration time, monitored using Fourier transform infrared spectroscopy. We show that the concentrations of electrolyte (LiBr) and proton source (dimethylurea) can be reduced while maintaining high reaction efficiency. We also report the reduction of 1-aminonaphthalene, which has been used as a precursor to the API Ropinirole. We find that our methodology produces the corresponding dihydronaphthalene with excellent selectivity and 88% isolated yield in an uninterrupted run of >8 h with a projected productivity of >100 g day^–1^.

## Introduction

**Figure 1:**
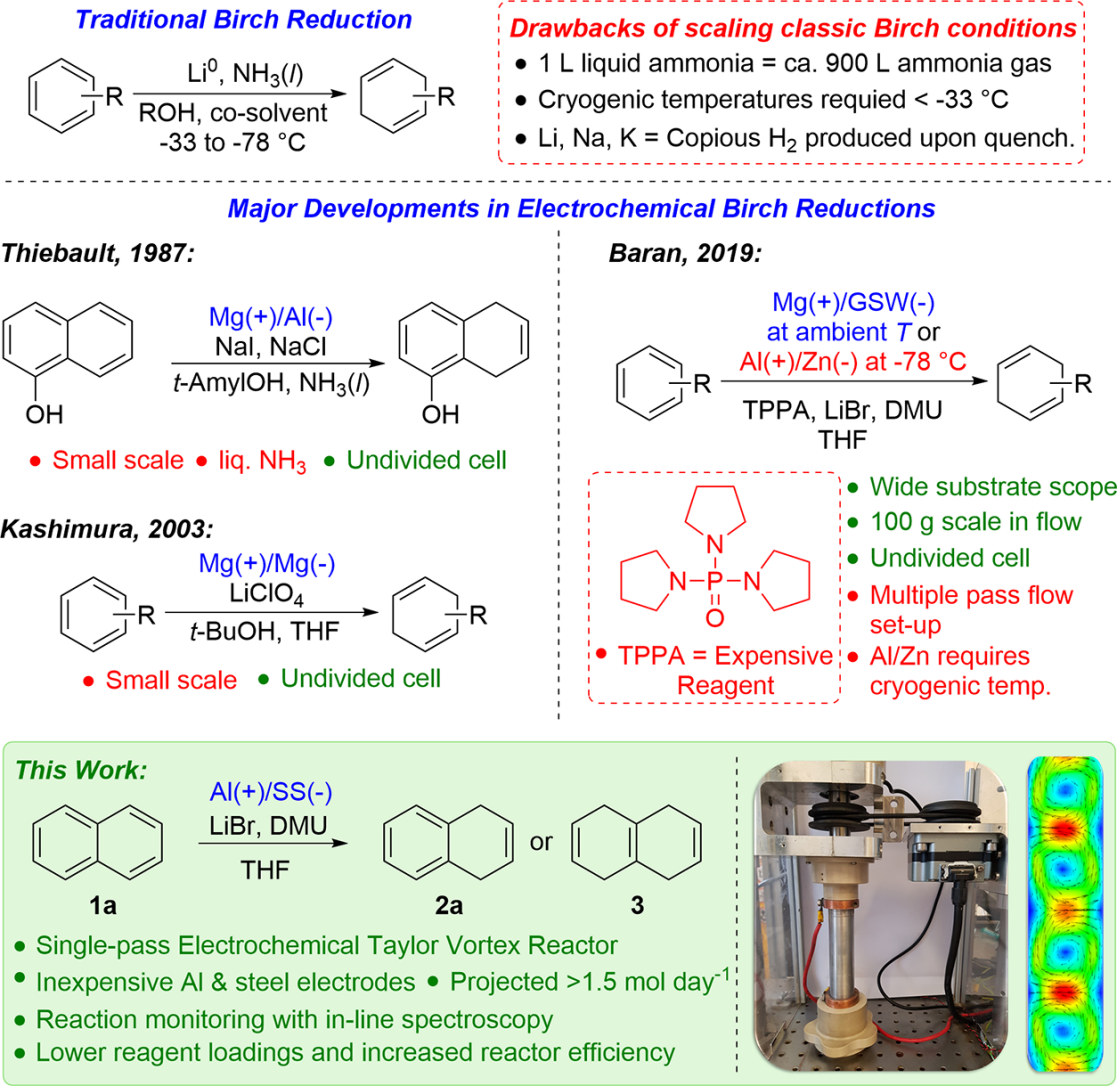
Schematic showing the traditional Birch reduction (top), together with the major developments in electrochemical variants of this reaction including our electrochemical Birch reduction approach described in this paper.

The dearomatization of arene moieties is of current interest, including the drive in medicinal chemistry to move away from the flat functionalities of aromatic rings and to explore alternative 3D scaffolds.^1−3^ Dearomatization can be achieved by utilizing a range of different methodologies,^4,5^ including oxidative methods,^6−8^ hydrogenation,^9^ and transition metal-mediated^10,11^ and nucleophilic addition approaches.^12^ Dissolving metal reduction, or Birch reduction, is one such method that allows for the direct reduction of aromatic rings to 1,4-dienes (Figure 1).^13^ The traditional synthetic methodology of this reaction requires the use of alkali metals (typically Li, Na, or K) dissolved in liquid NH_3_ which, in turn, necessitates cooling (typically <−33 °C) to maintain its liquid state.^14^ The harsh and challenging conditions of this methodology mean that it is not favored in industrial settings as it presents several safety challenges with copious amounts of ammonia required upon scale-up.^15,16^ While there have been several efforts to produce an ammonia-free Birch reduction, the methodologies have only been demonstrated on small scales^17−19^ and in some cases, they led to over-reduction of the Birch-type products.^20−22^ A recent report by Koide and co-workers demonstrated that a Birch reduction could be achieved by replacing ammonia with ethylenediamine in THF and lithium metal as the reducing agent.^15^ They scaled up this methodology to 61 g scale in batch. There have also been efforts to further develop a milder methodology by circumventing the need to use an alkali metal as the reducing agent; hence, there is a focus on photochemistry^23,24^ and electrochemistry. In these approaches, the alkali metal is effectively replaced by an electron supplied from either a photocatalyst or the surface of an electrode in an electrochemical cell.

Electrochemistry is an attractive methodology for this type of reduction because it uses electricity to drive the reaction, by relying on electrons transferred from an electrode surface in an electrochemical cell. Electrochemical methods can often enable access to reactive intermediates without the need for excessive or harsh reagents.^25−31^ Several key developments toward an electrochemical Birch reduction have been made (Figure 1); in 1987, Thiebault and co-workers described a single compartment electrochemical cell where they performed the electrochemical reduction of a range of different substrates using liquid ammonia, a dissolving magnesium anode and an aluminum cathode.^32^ In a similar manner, Bard and co-workers, in 1992, performed the reduction of C_60_ in liquid ammonia using Pt mesh electrodes.^33^ In 2003, Kashimura^934^ and co-workers reported the electroreduction of aromatics using magnesium electrodes and t-BuOH as a proton donor in THF. They discovered that the aprotic solvent THF was the optimal solvent for the transformation.^34^

These electrochemical examples only dealt with small-scale batch reactions, but more recently, Baran and co-workers reported an elegant methodology with the use of tripyrrolidinophosphoramide (TPPA, see Figure 1) as an additive inspired by Li-ion battery technology^16^ to prevent the plating out of Li metal, which can occur under electrochemical conditions.^34^ Their work involved two methodologies, one using a magnesium anode and galvanized steel cathode at ambient temperature and the second method using an aluminum anode and a zinc cathode with cooling to −78 °C. The first method was scaled up using a plate-type undivided cell operating in a circulating loop for 62 h to yield ca. 65 g of reduced TBDMS-protected *p-*cresol from 100 g of substrate.

Continuous flow processing techniques have many advantages over traditional batch setups for electrochemical reactions because interelectrode gaps are often small with a large electrode surface area relative to the volume of solution being processed, thereby boosting effciency.^29,35−42^ Many small-scale electrochemical reactor designs have been developed for flow chemistry over the past decade.^43−49^ One reactor type that had been under-utilized for synthetic electrochemistry is the Taylor vortex reactor.^50^ Recently, we reported an electrochemical flow Taylor vortex reactor^51^ that consists of a tubular electrode (anode—graphite) with a rotating cylinder (cathode—stainless steel) on the inside that serves as the second electrode. This reactor design builds on our successful photochemical vortex reactor designs reported previously.^52,53^ The gap between the electrodes is intentionally small (0.5–2 mm) so that Taylor vortices are formed in the annulus when the inner electrode is rapidly rotated. We have reported this use of the electrochemical Taylor vortex reactor with two electrochemical oxidation reactions, both of which gave high and tunable selectivity between two possible products. In this paper, we demonstrate the viability of using the electro-vortex reactor for electrochemical reductions involving sacrificial electrodes with a proof-of-concept study using the reductive dearomatization of naphthalene **1a** and of 1-aminonaphthalene **1b** which has been used as a precursor to the API Ropinirole.

## Results and Discussion

The electrochemical Taylor vortex reactor is based on the Taylor–Couette flow principle and consists of a static outer cylinder (*R*_o_ = 11 mm) and rotating inner cylinder (*R*_i_ = 9.5 mm), which act as the electrodes of the cell. There is a narrow gap of 1.5 mm width between the two cylinders that forms the annulus in which the reaction takes place. The internal volume of the annulus is 17.8 mL. A PEEK cap and base provide insulating seals between the two cylinders. The cap and base have ports to allow the reaction mixture to flow in and out of the reactor.

The reactor design allows for the formation of the axisymmetric toroidal vortices, known as Taylor vortices, that emerge when the rotation speed (Ω) of the inner cylinder is increased above the so-called critical rotation Reynolds number *Re*_cr_ = ρ*U_Ω_d*/μ (known also as Taylor number), where ρ is the density of the fluid, *U_Ω_* is the linear speed of the inner cylinder, *d* is the gap width, and μ is the dynamic viscosity of the fluid. The critical Reynolds number depends strongly on the radius ratio η = *R*_i_/*R*_o_, which is 0.8636 for the present reactor design. This value leads to the critical Reynolds number 109 according to the experiments reported by Di Prima and Swinney. Thus, the ratio between the actual Reynolds *Re*_Ω_ number and the critical Reynolds number *Re*_cr_ should be above 1 to allow for the formation of the Taylor vortex flow instability, which has a laminar regime. The continuous increase of the inner cylinder rotation speed leads to an increase in the number of the vortices and a progression of the Taylor vortex flow instability to become turbulent above a second critical value *Re*′_cr_. The Reynolds number ratio *Re*_Ω_/*Re*_cr_ of the turbulent Taylor vortex flow is around 20, according to the work of Di Prima and Swinney.^54^ Moreover, the increase in the rotation speed of the inner cylinder is expected to increase mixing efficiency within the gap and enhance the electrochemical reaction.

**Figure 2:**
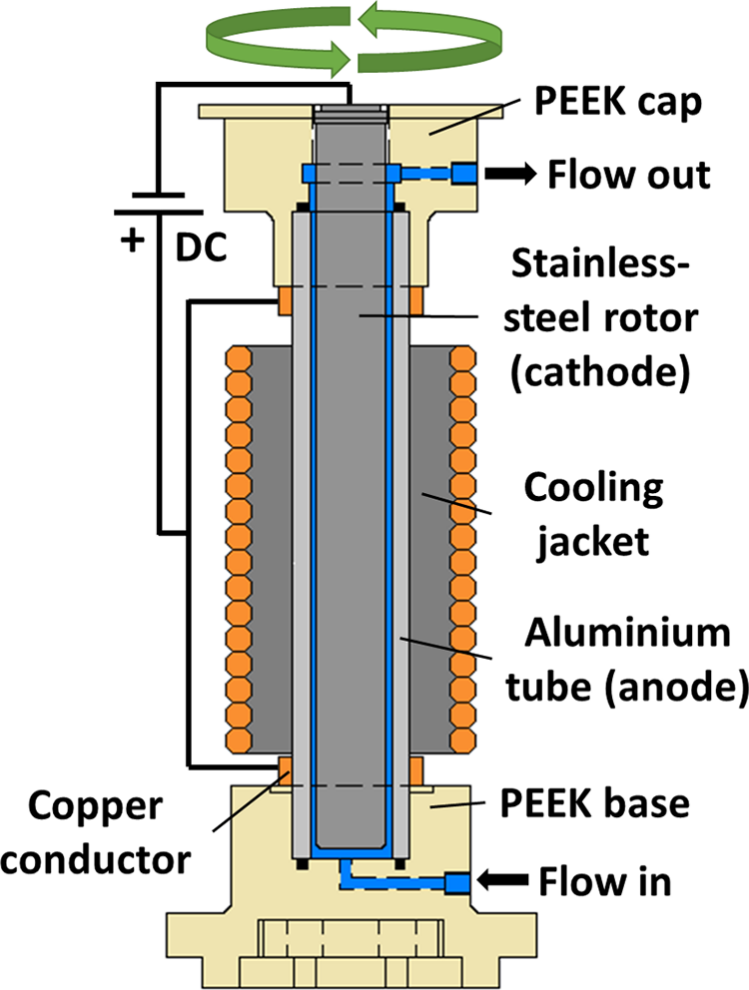
Simplified cross-section of the reactor, showing the reaction solution in blue. The cooling jacket consists of an aluminum block with a copper coil wrapped around. The motor and brush assembly, which provides the electrical connection to the rotor, which was completely smooth, is not shown explicitly. The reactor was only operated in the vertical orientation.

Our original report,^51^ focused on electrochemical oxidation, used a graphite tube as the outer anode of the reactor. Here, however, as a sacrificial anode is required for this type of reduction reaction, other materials were considered for the electrodes; indeed, when the reduction reaction was run with the graphite anode, no reaction took place. We opted for an aluminum anode as it is a commonly used sacrificial electrode and is relatively inexpensive, inert, and easy to machine, making it ideal for use in our reactor design. Stainless steel was used for the rotating inner cathode. A schematic of the reactor is depicted in Figure 2, and a full piping diagram showing the overall setup is detailed in the Supporting Information. Unpublished experiments to measure the residence time distribution in our original electrochemical Taylor vortex reactor with a carbon outer electrode^51^ indicated that the mean residence time was only slightly longer than the value estimated by dividing the reactor volume by the volumetric flow rate. The lengthening of the time may well be due to the adsorption of the Rose Bengal dye used in the experiment to the porous carbon electrode. It seems reasonable to suppose that a similar residence time distribution will apply to the reactor in this paper. Tachograph measurements on our belt drive system indicated that the actual rotational speed at the setting of 4000 rpm was within ±20 rpm of the set point.

**Figure 3:**
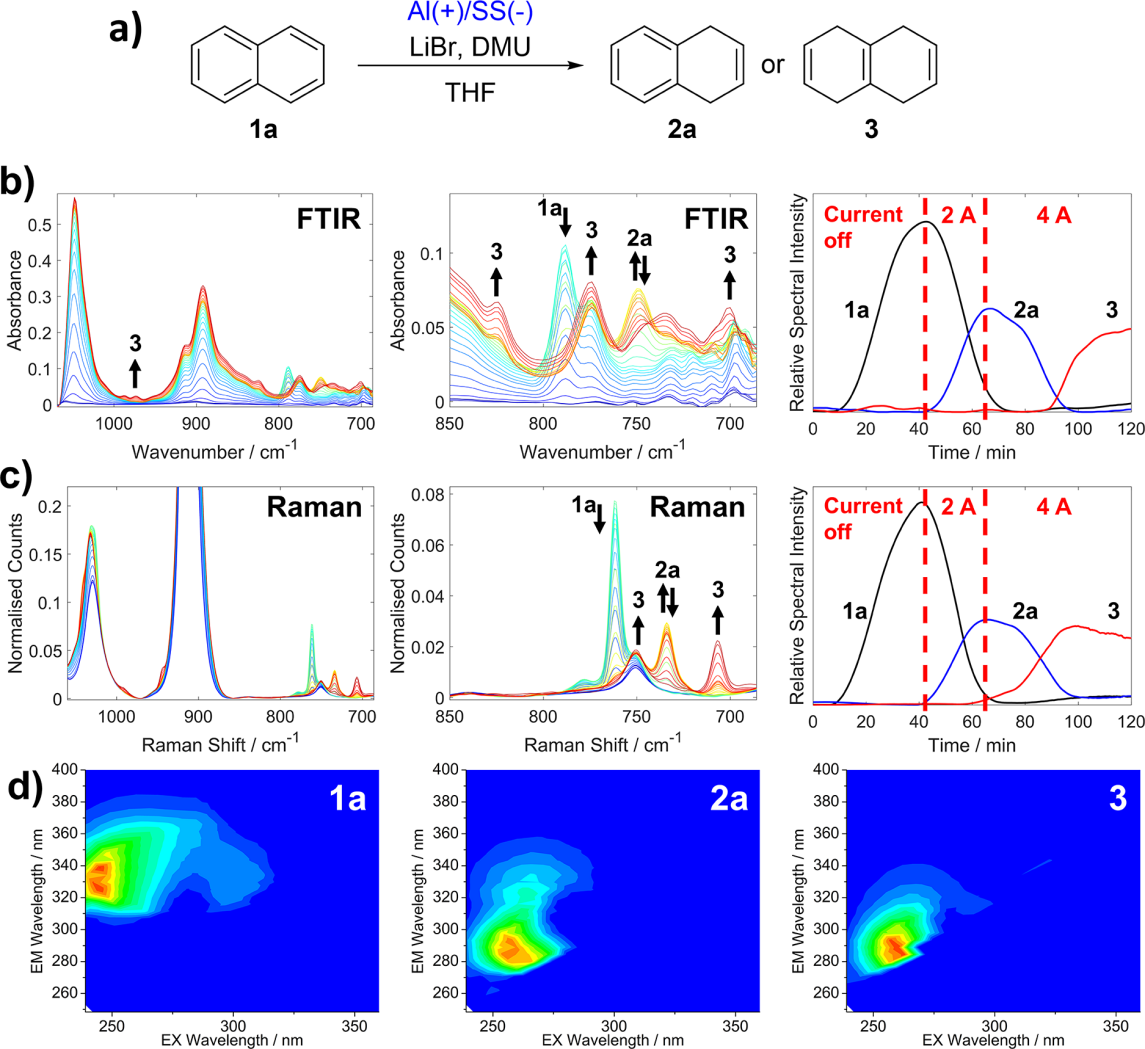
Process monitoring spectra for the continuous two-stage Birch reduction of naphthalene shown schematically in (a) with 18 mL reactor volume and a 1.75 mL/min flow rate (b) illustrates the FTIR data: Left and middle, solvent-subtracted inline FTIR spectra with peaks relating to **1a**, **2a,** and **3** beginning from the time that the fluid flowing through the reactor was switched from pure THF to the reaction mixture. The large peaks observed at ca. 890 and 1050 cm^–1^ are due to the changes in the THF spectrum associated with the addition of LiBr, and the band relating to **3** at 770 cm^–1^ overlaps with that of DMU. The curves on the right show the spectral intensities which are related to concentrations of **1a**, **2a,** and **3** at different currents. The curves are derived from the FTIR data after deconvolution with multivariate curve resolution. (c) Corresponding Raman data; left and middle, spectra with peaks relating to **1a**, **2a**, and **3**. The peaks at ca. 900 and 1025 cm^–1^ are due to THF as well as the small peak at ca. 750 cm^–1^ which overlaps with **3**. The curves on the right show the Raman monitoring of **1a**, **2a,** and **3** taken from spectral intensities after deconvolution with multivariate curve resolution. (d) EEM spectra of **1a**, **2a,** and **3** taken from samples collected at 40, 63, and 117 min into the operation of the reactor, respectively. The EEM spectra consist of a two-dimensional contour plot of excitation vs emission with the normalized fluorescence intensity shown by different colors (blue: low intensity, red: high intensity) which together provide the so-called “molecular fingerprint” of the compound (2-D and 3-D plots of EEM spectra are compared in Figure S20 in the Supporting Information).

With the electrode materials chosen, we began exploring conditions for the reduction of **1a** with the aim of selectively producing **2a** in high yield with a single pass through the reactor (Figure 3a). Our starting point was to repeat conditions from Baran’s report, that is, **1a** in THF with LiBr (7.5 equiv) as the electrolyte, DMU (*N*,*N*-dimethylurea, 3 equiv) as the proton source, and TPPA (10 equiv). Under these conditions, **1a** was consumed and the selectivity was promising; however, repeatability was poor as the reactor could not be operated for more than 20–30 min, at which point blockages were encountered due to the build of a black tar-like substance (see Supporting Information for further details). The omission of TPPA allowed the reaction mixture to be passed through the reactor without blockage; however, the desired current could not be achieved without increasing the amount of LiBr/DMU (see Supporting Information for further details). We initially found that **1a** in THF with 3 M LiBr and 12 equiv of DMU gave stable reaction conditions. We noted that in some cases, when the reaction was performed under nonanhydrous conditions, a cloudy solution containing black/gray particles sometimes appears in the output stream, in agreement with previous reports.^16^ By contrast, when the solution is prepared anhydrously (see Experimental Section), the output stream appears clear and free of insoluble particles and the reactor operated stably, running without problems for extended periods, tested up to 8 h. During the initial scoping of this reaction in the electrochemical Taylor vortex reactor, we discovered that it takes significantly longer to reach steady state compared to our previous studies, where approximately three reactor volumes were sufficient. In previous cases, we employed relatively dilute solutions but, due to the higher concentration of LiBr and DMU in this reaction mixture, the overall solution was relatively viscous in comparison to those studied before. Therefore, we chose to monitor the reaction using inline Fourier transform infrared (FTIR, Figure 3b) and Raman (Figure 3c) measuring at the reactor outlet, allowing us to follow, in real time, the consumption of the DMU and **1a** along with the appearance of **2a**/**3**. FTIR and Raman were used simultaneously to exploit their unique advantages, for example, the sensitivity for monitoring polar vibrational modes, particularly those of DMU (see SI), while Raman is more effective for the polarizable vibrations of the substrate and products. The Raman also facilitated the detection of species with vibrational modes <700 cm^–1^, a region needed to reliably monitor the production of **2b**, as discussed below. With these live data, we were able to monitor the equilibration of the reactor in real time to ensure that a steady state was achieved before taking samples (Figure 3).

In addition, steady state at-line absorbance–transmission fluorescence excitation and emission matrix (A-TEEM) spectra were taken of the three stages of the process (i.e., at steady states of **1a**, **2a,** and **3**). The A-TEEM technique allows us to simultaneously measure the absorption, transmission, and fluorescence of a sample in a matter of seconds and to construct a 3D “fingerprint” or spectral matrix unique to each sample, by correlating excitation and emission wavelengths to fluorescence intensity; this, in turn, can be used to monitor progress of the reaction (Figure 3d). The distinct excitation emission matrix (EEM) obtained from the reactor outflow demonstrates the characteristic changes associated with the dearomatization of the substrates. During the reduction, the emission energies of the compounds increase, and initially, there is large shift to a higher energy emission as **1a** is reduced to **2a**. However, there is a much less pronounced shift as **2a** is reduced to **3**. The combination of these three process analytical techniques (PAT) proved to be a powerful tool for monitoring the electrochemical reduction in our reactor.

After initial optimization (see Supporting Information for further details), we found that the reaction proceeded better in the absence of TPPA. With a flow rate of 1.75 mL min^–1^ and a current of 520 mA, we obtained high conversion of **1** (85%) with 84% yield of **2a** and 1% of **3**; that is, >98% selectivity toward the single reduction product **2a**. The viscosity of the reaction mixture was found to be ca. ×5.5 greater than that of neat THF and high viscosity is clearly undesirable for a flow process. We found that the concentration of LiBr contributed most to the viscosity and decided to investigate the effect of reducing the concentration of DMU and LiBr. Reducing the concentration of LiBr and DMU by half (1.5 M LiBr, 6 equiv DMU) and to a quarter (0.75 M LiBr, 3 equiv DMU) of the original amount led to only a slight decrease in the yield of **2a** to 59 and 61%, respectively, while maintaining high selectivity. The voltage required to maintain the 520 mA current varied for each of the three different concentrations of LiBr (see Supporting Information for further details). Interestingly, the 1.5 M LiBr solution required the lowest voltage (3.0 V versus 3.2 V for the 3 M solution), while the 0.75 M solution needed the highest voltage at 4.6 V. In addition to the variation in voltage and yield, a difference in the time required for the reaction to achieve a steady state output was also observed (see Supporting Information for further details). When using the less concentrated solution, the time to reach steady sate (ca. 40 min) was approximately half that of the higher concentration (ca. 80 min). Given this variation in equilibration time, we postulated that the variation in viscosity could yield a difference in the mixing efficiency for the three solutions. Indeed, the variation in viscosity affects the rotation Reynolds number, and thus, the higher concentration is expected to lead to a lower *Re*_Ω_ and consequently a slow mixing process and a higher equilibration time (e.g., the less concentrated solution at 4000 rpm inner cylinder rotation speed has a *Re*_Ω_ = 8300, whereas the higher concentrated solution at the same rotation speed has a *Re*_Ω_ = 2700). In addition, experiments in the literature showed that the viscosity increase results in a decrease of the number of vortices present in the reactor.

Computational fluid dynamics (CFD) is used to support both the experimental findings, theoretical explanation, and mixing efficiency hypothesis. The CFD simulations were conducted using the commercial software ANSYS-Fluent 2022R1. The modeled electrochemical Taylor vortex reactor was two-dimensional and symmetric with a mesh size of 146k nodes (see Supporting Information for further details about the validation of the CFD with the experiments from the literature). A species of a mass fraction of 0.1 that has the same properties of the solution is injected from the inner cylinder to model the electrochemical reaction. Two flow regimes are investigated, laminar and turbulent, to assess their effects on the mixing efficiency (equilibration time). For the laminar case, the inner cylinder rotation speed is set at 100 rpm for the 0.75 and 1.5 solutions and 200 rpm for the 3 M solution to ensure a Reynolds number ratio above 1. For the turbulent case, the inner cylinder rotation speed is set at 4000 rpm for the solutions, which guarantees a Reynolds number ratio above 20; see Table 1.

**Table 1:** Summary of the Modeled Cases in the CFD

solution	viscosity (kg m^–1^ s^–1^)	flow rate (mL min^–1^)	rotation speed (rpm)	*Re*_Ω_	*Re*_Ω_/*Re*_cr_
0.75 M	0.00069	1.75	100	200	1.9
			4000	8300	76.11
1.5 M	0.00092	1.75	100	200	1.48
			4000	6500	59.17
3 M	0.00253	1.75	200	100	1.22
			4000	2700	24.35

**Figure 4:**
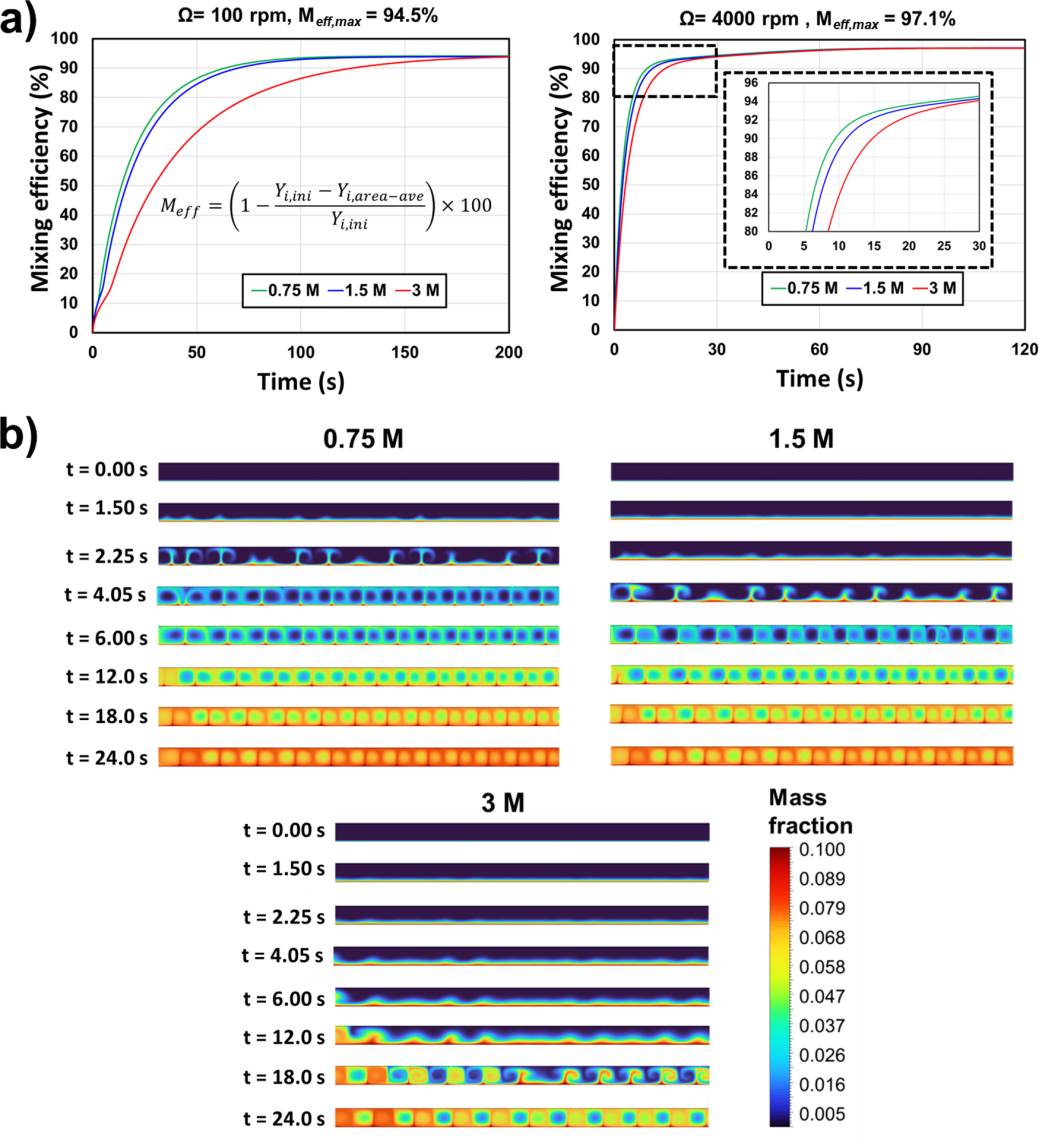
Results of a CFD investigation of the effect of the increasing viscosity of the reaction mixture with increasing concentrations of LiBr. (a) Plots of mixing efficiency over time for three solutions (0.75, 1.5, and 3 M) of increasing viscosity (left) at 100 rpm for and 200 rpm for the most viscous 3 M and (right) at 4000 rpm. (b) CFD simulations showing the formation of the vortices and their transport within the reactor gap over time.

The CFD results (Figure 4a) revealed that the equilibration time (when the mixing efficiency reaches a steady state) increases as the viscosity of the solution increases. This effect occurs both at laminar (100 and 200 rpm) and turbulent (4000 rpm) regimes. However, at high rotation speed (turbulent Taylor flow), the equilibration time is lower than that at low rotation speed (laminar Taylor flow). This is due to the increase in the momentum of vortices under turbulent regime, which contributes to the quick mixing of the solution, and consequently low equilibration time is achieved. Figure 4b shows the transport of the species that are a good tracer of the Taylor vortices for the different solutions and demonstrates the delay in the equilibration time when the solution is more viscous. The reason behind this is due to the increase in the shear stress (flow resistance force) at higher fluid viscosity, which delays the momentum transfer from the rotating cylinder to the solution in the gap. Thus, the Taylor vortex formation is also delayed. Moreover, one can notice (Table 1) that at the turbulent regime (4000 rpm), the Reynolds number ratio (*Re*_Ω_/*Re*_cr_) of the higher viscous solution (3 M) is almost three times lower than that of the less viscous solution (0.75 M).

The effect of rotation speed was studied using the 1.5 M solution, which, surprisingly, revealed that there was little difference in the yields of **2a** and **3** at the different rotation speeds studied, suggesting that any mixing is sufficient for the chemistry (see Supporting Information for further details). However, the study did reveal that operating the reactor without rotation was detrimental both to the overall yield and to the selectivity of the reaction. Furthermore, with no rotation, the voltage required to reach the desired current was significantly higher (ca. 8.5 V at 0 rpm vs ca. 3.0 V with rotation).

The productivity of the reaction could be increased by simultaneously increasing the flow rate and current; however, higher currents necessarily required higher voltages leading to increased temperatures of the reactor. Therefore, an aluminum cooling jacket with a copper coil fed by a recirculating chiller was used to maintain a stable temperature in the reactor. We found, in terms of conversion and selectivity, that the reaction worked best when the temperature was maintained in the range of 10–30 °C; see Table 2. With the temperature maintained at approx. 25 °C, the selectivity of **2a** could be as high as 98% (Table 2, entry 1), and when the applied current was doubled, the selectivity could be inverted to produce **3** in 94% yield (Table 2, entry 2). At higher flow rates and currents, it appears that the selectivity of **2a** decreased slightly from 98% (with 520 mA at 1 mL min^–1^, Table 2, entry 1) to 91% (with 1040 mA at 2 mL min^–1^, Table 2, entry 3) and then to 82% (with 2080 mA at 4 mL min^–1^, Table 2, entry 5). However, the selectivity for **3** remains approximately the same (94–96% Table 2, entries 2,4 and 6). With 2080 mA, the yield of **2a** (Table 2, entry 5) was projected to be delivered at 81 g day^–1^ (620 mmol day^–1^). For the double ring reduction at the highest current and flow rate (Table 2, entry 6), **3** was projected to be delivered at 100 g day^–1^ (770 mmol day^–1^).^55^

**Table 2:** Scaling of Current and Flow Rate to selectively produce **2a** and **3** in a single pass of the electro-vortex reactor^a^

entry	flow rate (mL min^–1^)	res. time (min)^b^	*I* (mA)	voltage (V)^c^	conv. of **1a** (%)^d^	yield of **2a** (%)^d^	yield of **3** (%)^d^	selectivity of **2**/**3** (%)	projected productivity,^e^ g h^–1^
1	1	17.8	520	3.5	96	94	2	**2–**98	1.04
2	1	17.8	1040	5.7	100	6	94	**3**–94	1.03
3	2	8.90	1040	5.9	97	88	9	**2**–91	1.92
4	2	8.90	2080	11.4	100	6	94	**3**–94	2.09
5	4	4.45	2080	11.1	94	77	17	**2**–82	3.37
6	4	4.45	4160	23.8	100	4	96	**3**–96	4.26

a Standard conditions: 4000 rpm, 0.14 M **1**, 1.5 M LiBr and 6 equivalents of DMU in THF.

b Res. time = residence time based on reactor volume of 17.8 mL.

c Average voltage measured at steady state.

d Conversion and yield determined by ^1^H NMR analysis.

e Projected productivity = [[conc. of **1a** × flow rate]/1000] × MW of **2a**/**3**] × 60 × % yield (% yield is expressed as a decimal). MW of **2a** = 130.19. MW of **3** = 132.21. Actual throughput was 300 mL of reaction mixture.

**Figure 5:**
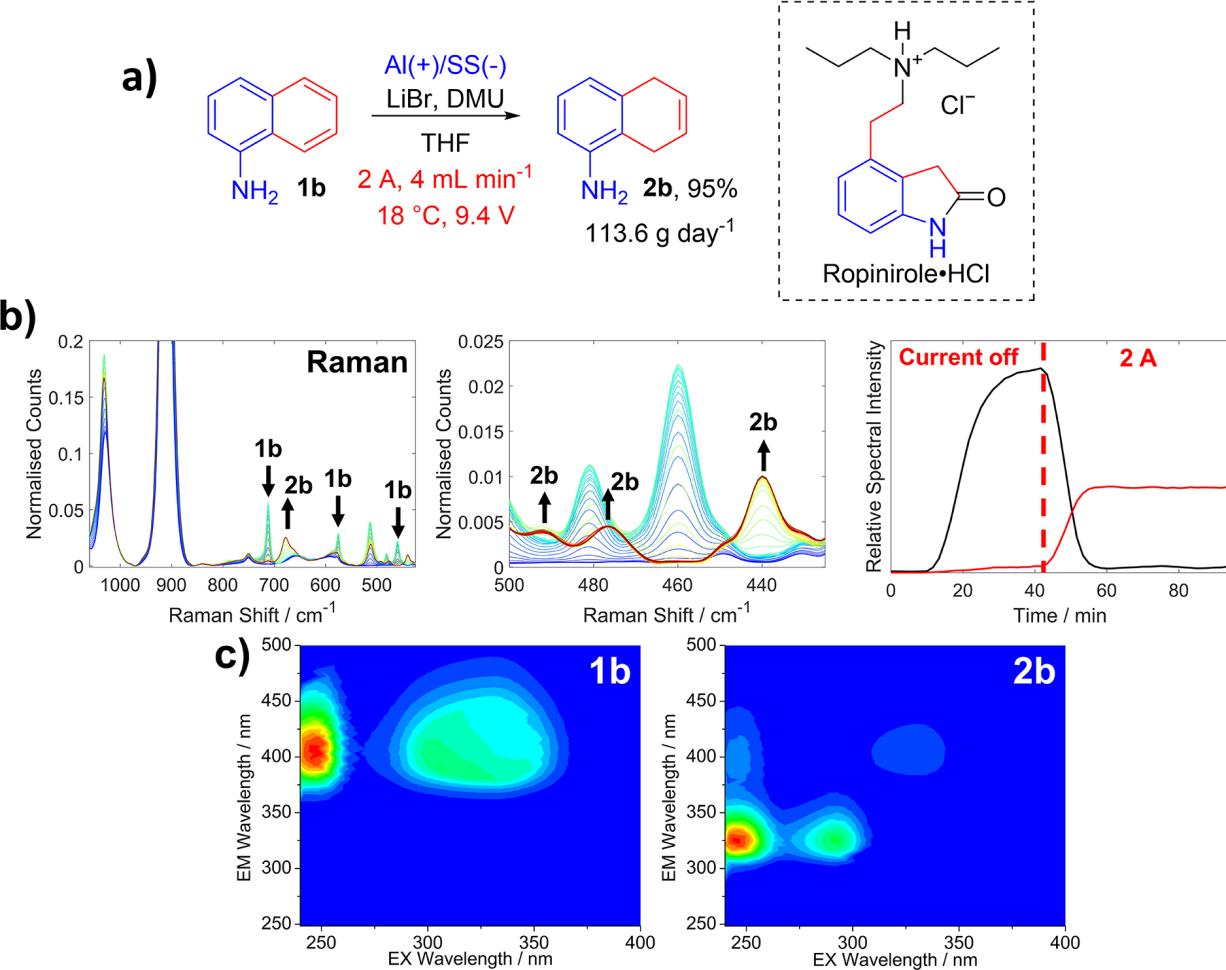
(a) Electrochemical Birch reduction of the naphthalene derivative **1b** previously used as a route to the API Ropinirole·HCl (showing the isolated yield after collecting 100 mL of the reaction mixture at the steady state); (b) left and middle, Raman Spectra showing peaks for **1b** and **2b**. The peak at ca. 520 cm^–1^ is due to DMU, and the peaks at ca. 900 and 1025 cm^–1^ are due to THF. (b) Right, intensity of **1b** and **2b** monitored using the 475 and 425 cm^–1^ vibrations, respectively. (c) EEM spectra of **1b** and **2b** taken from samples collected at 39 and 88 min into the operation of the reactor, respectively. Again, the normalized EEM spectra consist of a three-dimensional contour plot of excitation vs emission vs fluorescence intensity (blue: low intensity, red: high intensity).

We investigated the viability of the methodology on other naphthalene derivatives, in particular 1-aminonapthalene **1b** which is of interest because of its use in the synthesis of the API Ropinirole·HCl, developed by Harrowven and co-workers (Figure 5a).^56^ The reduction product **2b** was formed with high yield and selectivity, with only a trace of the over-reduction product, 5-aminotetralin, present. A sample (100 mL) was collected once the reactor was at the steady state, which was worked up to give an isolated yield of 95% for **2b** which equates to a projected productivity for **2b** of 114 g day^–1^ (782 mmol day^–1^). The conversion of **1b** was also followed with PAT (Figure 5b,c). In this instance, Raman spectroscopy and A-TEEMS proved to be the best option because the most prominent peaks for **1b** were outside the spectral window of FTIR (see Supporting Information). Nevertheless, the consumption of DMU could again be followed by FTIR and corresponded well with the consumption of **1b,** as shown in the Raman spectra. Additionally, in the A-TEEMS fingerprints, the dearomatization of **1b** was again accompanied by a blueshift in emission energy as **2b** was produced, displaying a distinct EEM signature that is characteristic of the product. This demonstrated the speed (∼10 s) at which A-TEEMS spectroscopy can be deployed to monitor a reaction, delivering a spectral signature that is easier for an operator to interpret than the more traditional vibrational spectroscopic approaches, such as Raman. Figure 5c also demonstrates that the A-TEEMS was sensitive enough to detect the residual **1b** (<5%) in solution that was not converted to **2b**.

We then ran the reduction of 1-aminonaphthalene, **1b,** continuously for >8 h (until our supply of **1b** was exhausted). A 2.0 L solution of reaction mixture containing 40 g 1-aminonapthalene was processed in the electrochemical Taylor vortex reactor over a continuous period of 8 h 20 min at a flow rate of 4 mL min^–1^. ^1^H NMR spectra were consistent with >90% selectivity toward the desired reduction product, and an isolated yield of 88% was obtained (35.8 g, 0.25 mol). Accompanying this, we analyzed the loss of mass and the change in the internal diameter of the tubular Al sacrificial electrode (see Supporting Information for data). The total mass loss from the Al electrode was 6.73 g (0.25 mol). Therefore, there was an approximate 1:1 molar ratio of **2b**:Al, slightly higher than the 1.5:1 ratio of **2b**:Al^3+^ that would be expected for 100% efficiency. We also carefully measured the effect of Al loss on the dimensions of the electrode. Initially, the internal diameter of the Al electrode was 22.0 mm (corresponding to a 1.5 mm gap size); after running the reaction for >8 h, we cut the tube in half (through the cylinder cross-section at the midpoint of the length of the tube) and took multiple measurements of the diameter of bore of the Al electrode (see Supporting Information). These showed that, on average, the gap size had increased by 0.2 mm over the 8 h period. This increase in gap size appeared slightly larger at the bottom of the tube (where the reaction solution first enters) than at the top of the tube (where the reaction solution exits, see Supporting Information).

This erosion of metal corresponds to an increase of 0.4 μm per min, and so we would expect the initial gap size (1.5 mm), to approximately double over a period of 62.5 h. A feature of this reactor Taylor vortex design is that, for a fixed gap size, the ratio of reactor volume to electrode surface area remains approximately constant as the diameter of the reactor is increased. This means that one might expect that the rate of erosion of the sacrificial electrode would be similar to that which we observed if the reactor were to be scaled up by increasing its diameter.

In these experiments, the starting solution was reddish in color and the product solution emerged from the reactor was black in color. This color was due to a solid suspension which could easily be removed by centrifuging. The black residue contained some DMU (by NMR) and 2.1% Li and 8% Al (by ICP-OES).

## Conclusions

The single-pass electrochemical reduction of naphthalene has been developed in a continuous flow vortex reactor using inexpensive aluminum and stainless-steel electrodes. Using 1.5 M LiBr as the electrolyte and DMU as the proton source, excellent selectivity was obtained between the two possible products. The single ring-reduced product **2a** was obtained in quantities up to 4 g with a projected productivity of ca. 80 g day^–1^, and the double ring-reduction product **3** could be obtained in quantities up to 5 g with a productivity of ca. 100 g day^–1^. We found that rotation of the reactor was necessary to obtain high selectivity with the added benefit of a reduced voltage to deliver the required current. The amount of LiBr and DMU processed could be reduced, while still maintaining a high efficiency reaction with high selectivity. Like all sacrificial electrodes, the aluminum will be corroded by the reaction and will produce particles in the reaction mixture that represent a blockage risk, especially in narrow flow paths. However, we have demonstrated that the reactor can be run for over 8 h without blockage, with constant performance in terms of selectivity and yield, and with an average increase in the annular gap between the electrodes of only 0.2 mm. Furthermore, because the metal is lost from the inner surface of the cylindrical electrode and all seals are on the outer surface, the loss of aluminum does not cause any leaks from the reactor. Eventually, the electrode will need replacing but the simple design of our reactor makes the replacement quite straightforward. Finally, the methodology was applied to a derivative of naphthalene **1b** which is used in the route to the API Ropinirole. The corresponding dihydronaphthalene **2b** was produced in excellent yield (90%) over 8 h of continuous operation, with a projected productivity of >100 g day^–1^. All of our reactions were monitored by on-line PAT (FTIR and Raman), and we have demonstrated what we believe to be one of the first applications of at-line A-TEEMS spectroscopy for fingerprinting the products of organic flow chemistry.

## Experimental Section

### General Experimental

Reagents were obtained from commercial sources and used as received unless stated otherwise. LiBr was obtained as anhydrous grade. THF was dried over activated alumina (ca. 8 ppm H_2_O). ^1^H NMR spectra were recorded at 400 MHz, and ^13^C NMR spectra were recorded at 100 MHz using a Bruker AV400. ^1^H NMR multiplicities are reported as s (singlet), d (doublet), t (triplet), and m (multiplet). The power supply was operated in constant current mode, where the desired current was set for each experiment and the maximum voltage was set to 30 V. The electrochemical vortex reactor was described previously and was used without change except for the static outer electrode materials.^51^ The reaction solution was delivered by a JASCO PU-4180HPLC pump. The solution was removed from the reactor using a Cole Parmer Masterflex L/S peristaltic pump. All pipe work was 1/8″ O.D. and 1/16″ I.D. PTFE tubing with Idex super flangeless fittings. The electrical output was delivered using a Keithley 2260B-30-72, 720 W power supply. Rotation to the reactor was provided using an Oriental Motor BLM5120P-A round shaft brushless motor with a BMUD120-C control box. The motor was connected to the reactor using a rubber belt and was checked for periodically for slippage using a Tachometer. The reported rpm in the study describes the set point of the motor controller, which was found to be ±20 rpm with the Tachometer. The recirculating chiller used in this study was a Julabo FL2503 filled with 1:1 ethylene glycol: water. The aluminum electrode was machined from 6082 aluminum and the steel electrode from 316 stainless steel.

### Spectroscopy Details

Inline FTIR monitoring was conducted using a Mettler Toledo ReactIR 702 L with a 1.5 m long silver halide fiber optic with a diamond element (Mettler Toledo DST FP-6-203-1.5). The spectra were collected using 8 scans at 10 s intervals. FTIR spectra were then post-processed by baseline-subtracting a first-order polynomial fitted in the 700–1080 cm^–1^ region and then block-averaging sets of 10 spectra together. Inline Raman monitoring was conducted using a Kaiser Optical Systems inc. RXN2 process Raman spectrometer equipped with a fiber optic attached to a Marqmetrix sapphire tipped Process Elite BallProbe 0.25. Raman spectra were collected using a 785 nm excitation laser with a power of 400 mW and a detector exposure time of 5 s with one scan, resulting in a spectrum every 10 s. To monitor **1a**, **2a**, and **3**, the spectra were processed using a first-order baseline subtraction in the 625–790 cm^–1^ region, and in the case of the monitoring of **1b** and **2b**, the spectra were baselined-processed using a Whittikar filter, in order to remove mild fluorescence. The Raman spectra were then block-averaged together in both cases in sets of 15 to produce the final spectra. A-TEEMS measurements were conducted using the Horiba Aqualog spectrometer, using a 1.5 mm path length Hellma Analytics three-windowed fluorescence cell. Absorbance spectra were collected between 248 and 800 nm with a resolution of 5 nm. The excitation emission matrices were collected using a CCD detector integration time of 0.1 s on the medium gain setting, with an excitation step size of 5 nm and an emission resolution of 3 nm. All spectra collected were processed against a THF solution of LiBr and DMU at equivalent concentrations to the mixture used for the reduction to obtain both an EEM and absorbance spectrum blank. Samples were collected from the reaction in flow after steady state was reached and then diluted by a factor of 100 before the measurements. The final excitation emission matrices obtained were then normalized by to their maximum value.

### General Procedures for Reductive Dearomatization of **1a**-**b**

#### Preparation of a Small-Scale Reaction Mixture

DMU (22.26 g, 152.8 mmol) was placed in a 500 mL round bottom flask and dissolved in 1:4 methanol:toluene (ca. 200 mL) and rotary-evaporated and dried further to remove and residual water, to remove any water, which yielded a fluffy white solid once dry. The solid was then further dried by hi-vacuum for 20 min to remove any residual solvent. LiBr (45 g, 518.2 mmol) was added to the flask, and then naphthalene (5.406 g, 42.2 mmol) was added to the DMU flask followed by the LiBr solution. Anhydrous THF (300 mL) was then added to the flask using a cannula, and then a balloon of argon was applied. The mixture was then sonicated until all the solids had dissolved.

#### Preparation of a Large Scale Reaction Mixture

LiBr (260.57 g, 3.0 mol) and 3 Å molecular sieves (20 g) were added to a 3 L round bottom flask and flame-dried. DMU (148.70 g, 1.7 mol) and 1-aminonaphthalene (40.09 g, 0.28 mol) were added, followed by anhydrous THF (made up to 2.0 L total volume) and sonicated until dissolution. The flask was then evacuated and backfilled with argon six times, using a Schlenk line with care so as not to evaporate the THF. After the final backfill with argon, a septum was applied followed by a balloon of argon.

#### Operation of the Reactor

The reactor was chilled to the desired temperature, and the motor providing rotation was started (set to 4000 rpm). The system was then flushed with anhydrous THF (approx. 100 mL) to remove any residual moisture. The input HPLC pump was set to the desired flow rate, and the output peristaltic pump was set to a value in a slight excess to the input flow rate to ensure that the reactor did not overfill. Once the temperature of the reactor reached equilibrium, the feed was switched to the reaction mixture, which was passed through the system at the desired flow rate until a steady state was achieved as shown by the inline monitoring (typically FTIR). Once at the steady state, the power supply was switched on and the current applied. The reactor was allowed to reach a steady state again, determined by inline monitoring, before sample collection. Once the experiment was complete, the power supply was switched off and the system was flushed with THF (ca. 100 mL) until the reaction components were no longer detected by the inline spectrometer. The system was then flushed with methanol liberating a dark residue, and this was continued until the outflow feed became clear (ca. 100 mL).

### Isolation

The collected sample was transferred to an appropriately sized round bottom flask, and the THF was removed by rotary evaporation. The residue was then re-dissolved in diethyl ether (200 mL) and transferred to a separation funnel. An aqueous solution of potassium sodium tartrate (0.5 M, 200 mL) was added and mixed, and the organic layer was collected. The organic layer was then washed with brine, dried over MgSO_4_, and then filtered and concentrated by rotary evaporation, yielding the isolated compounds for analysis. Further purification could be carried out by column chromatography using alumina and pentane/hexane as the eluent.

#### 1,4-Dihydronapthalene **2a**(57)

Isolated as a clear oil. ^1^H NMR (400 MHz, CDCl_3_): δ 7.18–7.11 (m, 4H), 5.95–5.92 (m, 2H), 3.42–2.40 (m, 4H). ^13^C NMR (100 MHz, CDCl_3_): δ 134.3 (2 × C), 128.5 (2 × CH), 126.0 (2 × CH), 124.8 (2 × CH), 29.8 (2 × CH_2_).

#### Isotetralin **3**(16)

Isolated as a white solid. ^1^H NMR (400 MHz, CDCl_3_): δ 5.73 (s, 4H), 2.54 (s, 8H). ^13^C NMR (100 MHz, CDCl_3_): δ 124.6 (4 × CH), 123.4 (2 × C), 31.0 (4 × CH_2_).

#### 1-Amino-5,8-dihydronapthalene **2b**(56)

Isolated as a reddish solid. ^1^H NMR (400 MHz, CDCl_3_): δ 7.00 (t, *J* = 7.7 Hz, 1H), 6.59 (d, *J* = 7.7 Hz, 1H), 6.55 (d, *J* = 7.7 Hz, 1H), 5.92–5.89 (m, 2H), 3.57 (s, 2H), 3.47–3.34 (m, 2H), 3.12–3.08 (m, 2H). ^13^C NMR (100 MHz, CDCl_3_): δ 144.0 (C), 134.8 (C), 126.7 (CH), 124.9 (CH), 123.3 (CH), 119.4 (C), 119.0 (CH), 112.5 (CH), 29.8 (CH_2_), 25.2 (CH_2_). HRMS (ESI) *m*/*z* calcd for C_10_H_12_N [M + H]^+^ 146.0964 found 146.0970.

## Supporting Information Available

Additional FTIR and Raman spectra and ^1^H and ^13^C NMR spectra for compounds **2a-b** and **3** (PDF)
