# Supplementary material for: High-Productivity Single-Pass Electrochemical Birch Reduction of Naphthalenes in a Continuous Flow Electrochemical Taylor Vortex Reactor
Source: Org Process Res Dev. 2022 Aug 24;26(9):2674–84. doi: 10.1021/acs.oprd.2c00108 (PMC9486933; doi:10.1021/acs.oprd.2c00108)
Supplement: Supplementary file 1 — op2c00108_si_001.pdf [file op2c00108_si_001.pdf]

Electronic Supporting Information

High Productivity Single Pass Electrochemical Birch Reduction of Naphthalenes in a Continuous Flow Electrochemical Taylor-Vortex Reactor

Darren S. Lee[a], Ashley Love[a], Zakaria Mansouri[b] Toby H. Waldron Clarke[a], David C. Harrowven[c], Richard Jefferson-Loveday[b], Stephen J. Pickering[b], Martyn Poliakoff[a], Michael W. George*[a]

a) School of Chemistry, University of Nottingham, University Park, Nottingham, NG7 2RD, UK

b) Department of Mechanical and Manufacturing Engineering, University Park, University of Nottingham, Nottingham NG7 2RD, U.K.

c) School of Chemistry, University of Southampton, Highfield, Southampton, SO17 1BJ, UK

*Email: mike.george@nottingham.ac.uk

Table of Contents

NMR Spectra.......2
Additional Experimental Data.......5
Extended Reactor Operation.......10
Examination of the sacrificial electrode following the > 8 hr run........12
Additional FTIR Spectra.......15
Additional Raman Spectra.......21
A-TEEM Spectroscopy.......22
Computation Fluid Dynamic (CFD) Details.......23

NMR Spectra

^{1}H NMR Spectra of compound **2a**

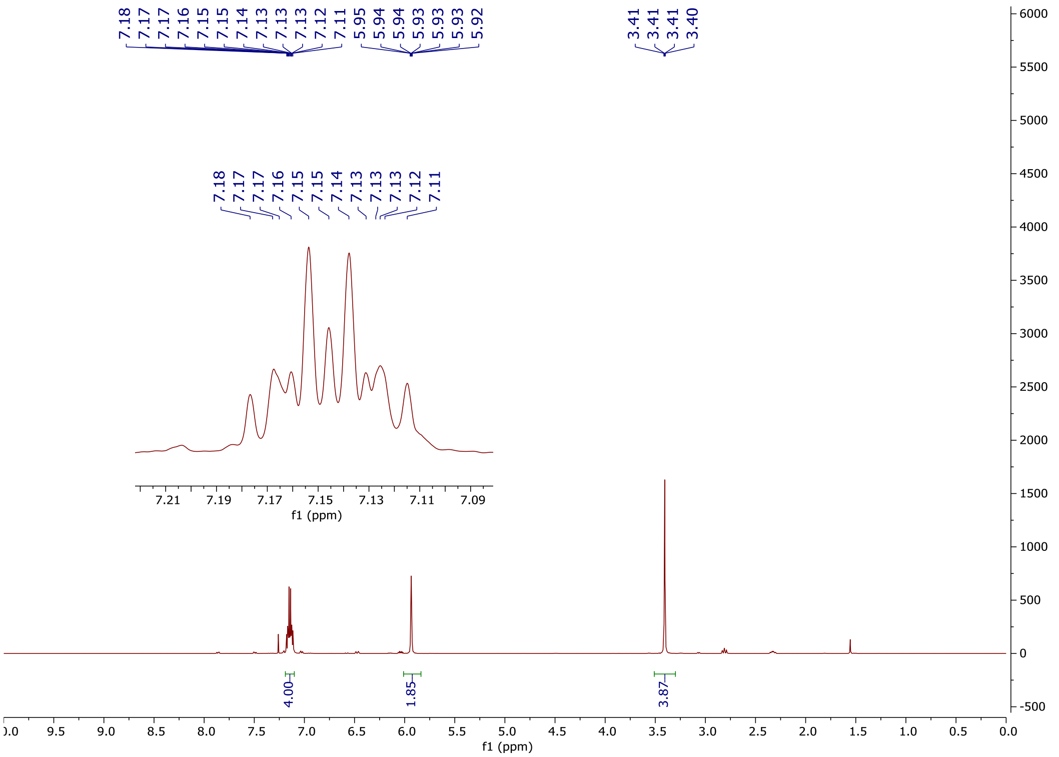

^{13}C NMR Spectra of compound **2a**

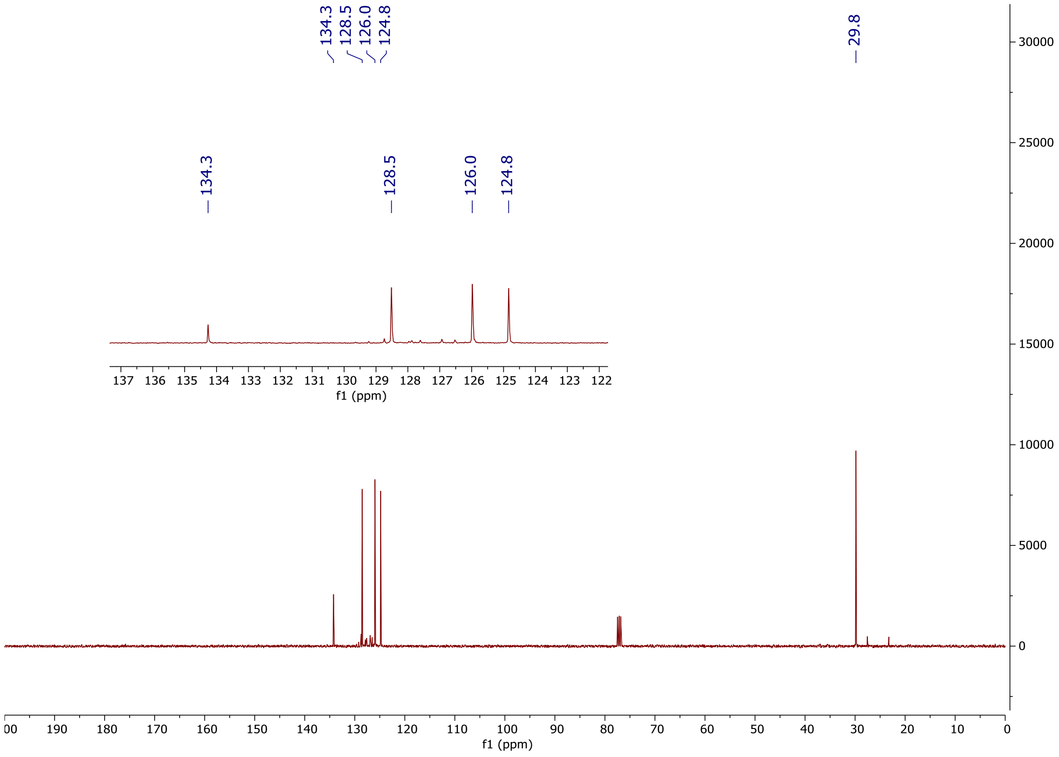

^{1}H NMR Spectra of compound **3**

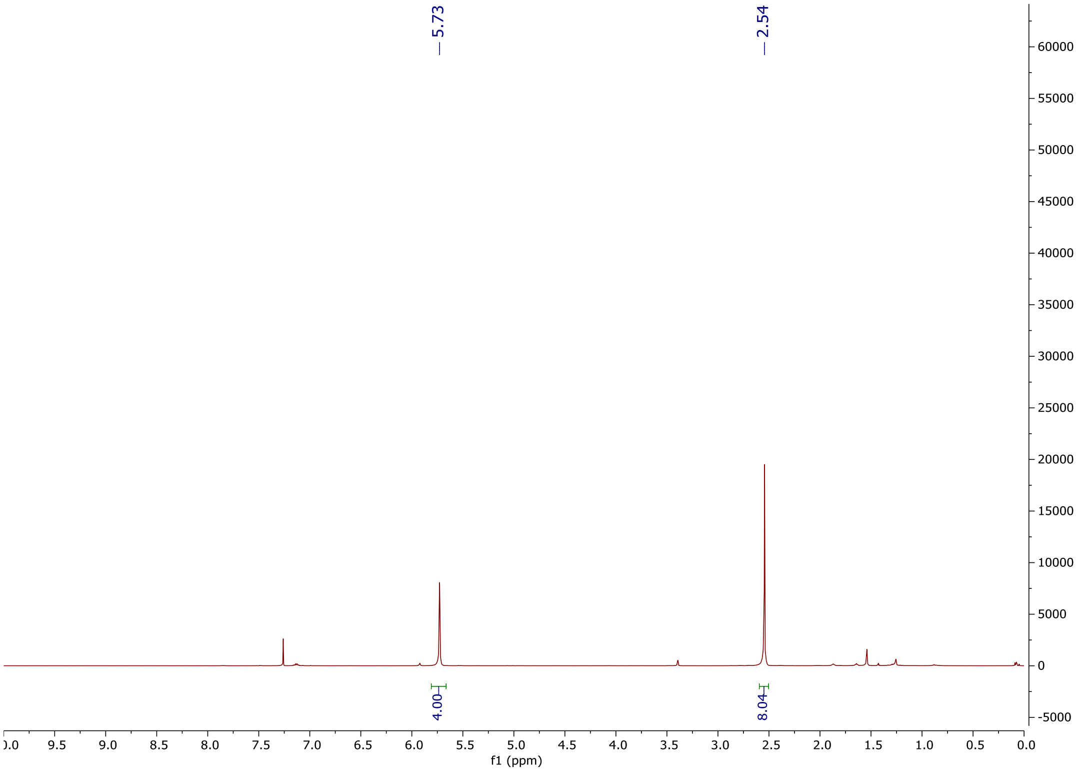

^{13}C NMR Spectra of compound **3**

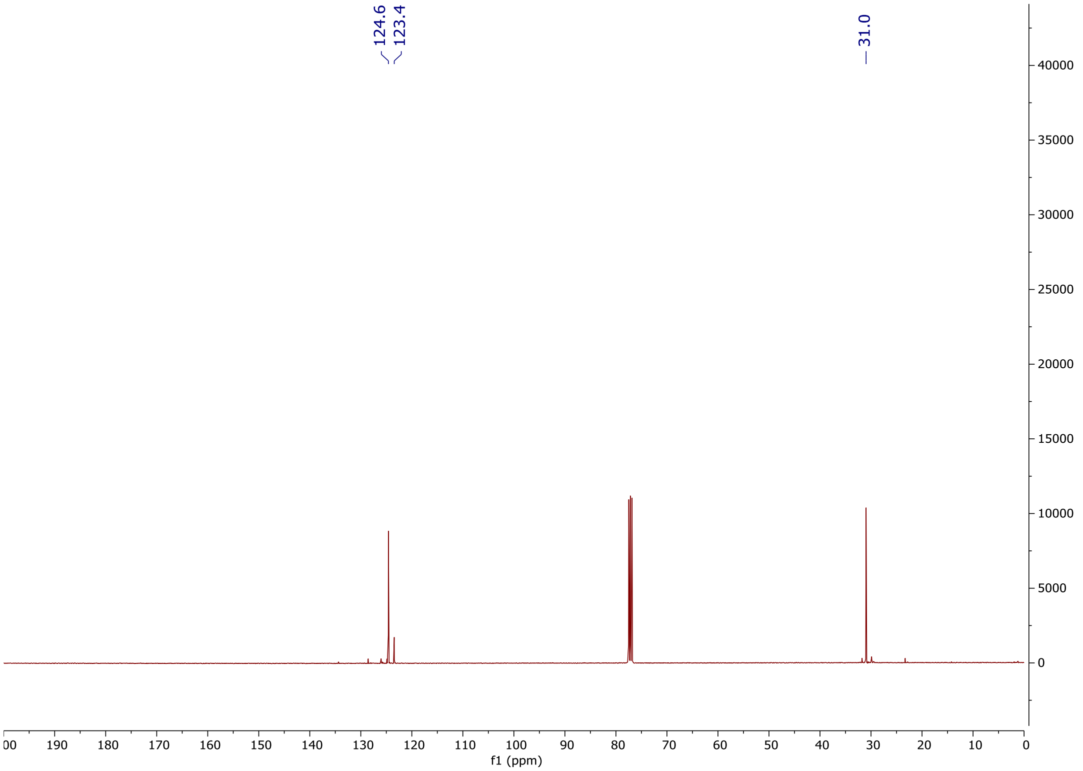

^{1}H NMR Spectra of compound **2b**

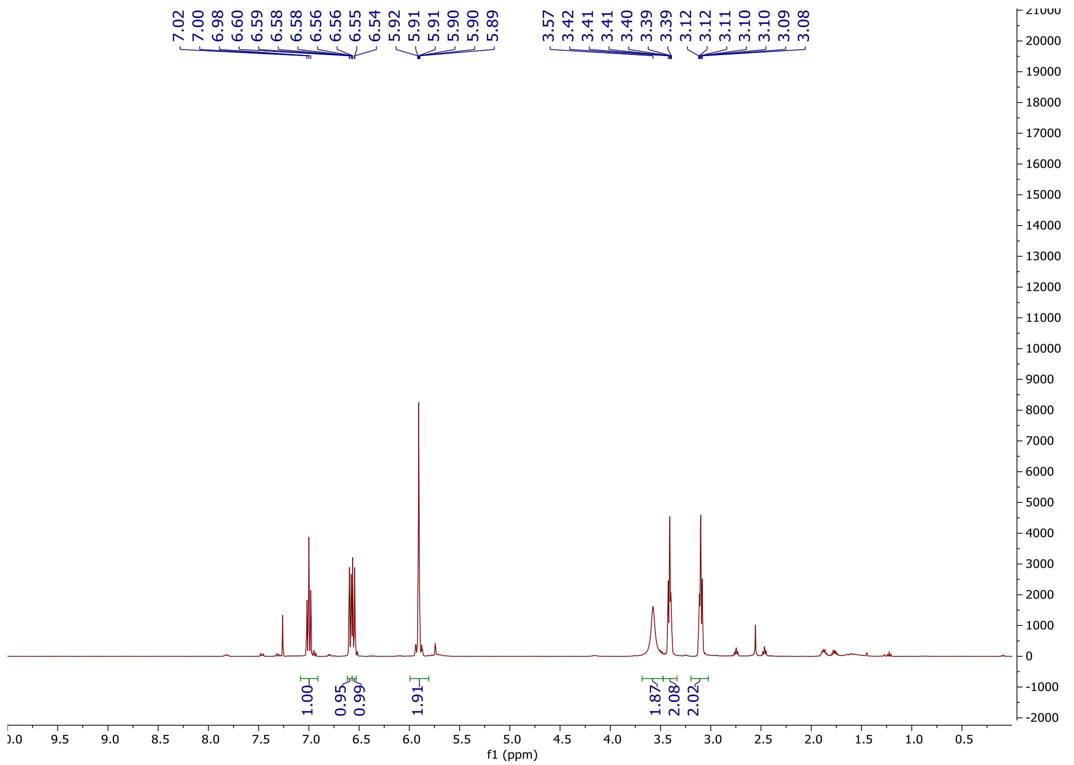

13C NMR Spectra of compound **2b**

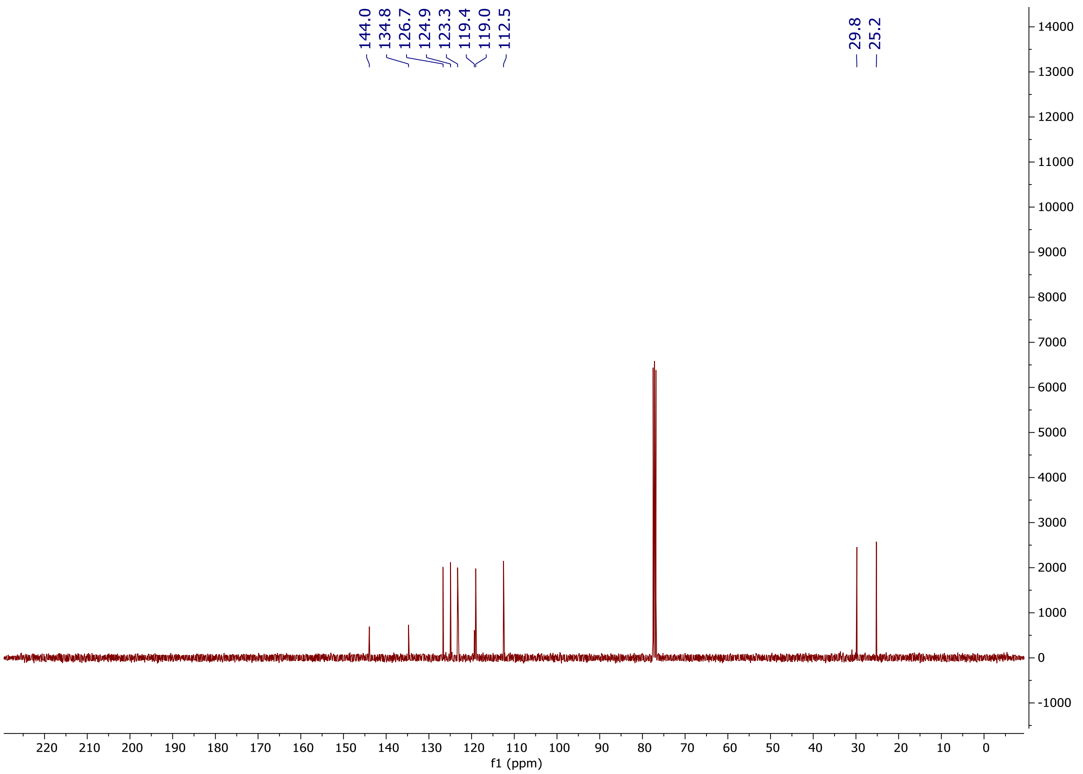

Additional Experimental Data

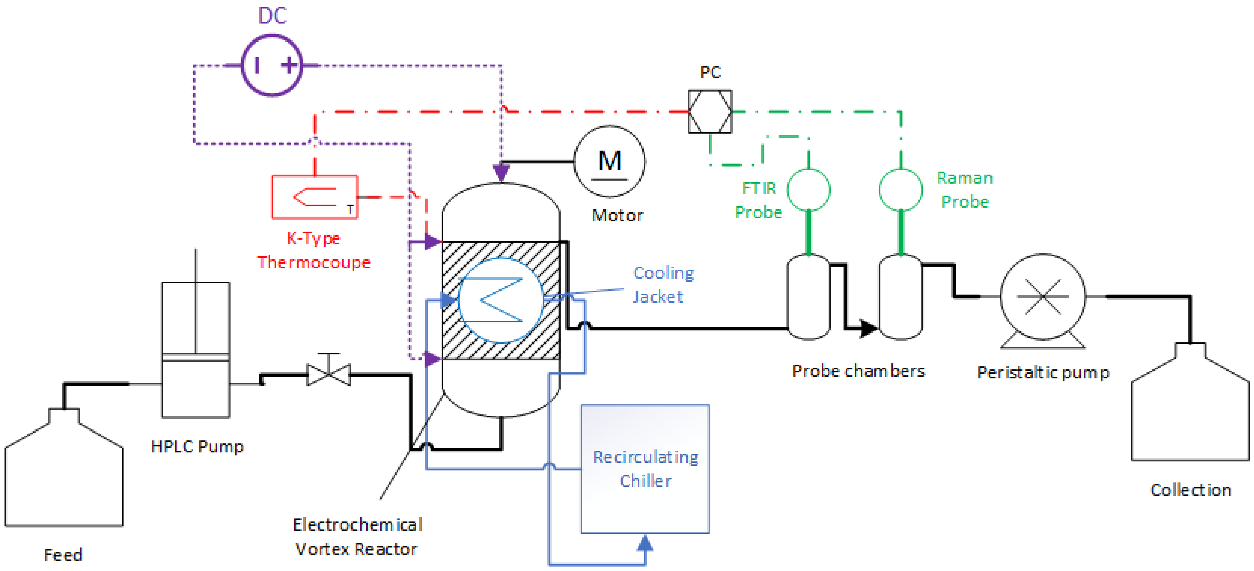

Figure S1. Piping diagram showing the system set-up, pumps, chiller and inline spectroscopy.

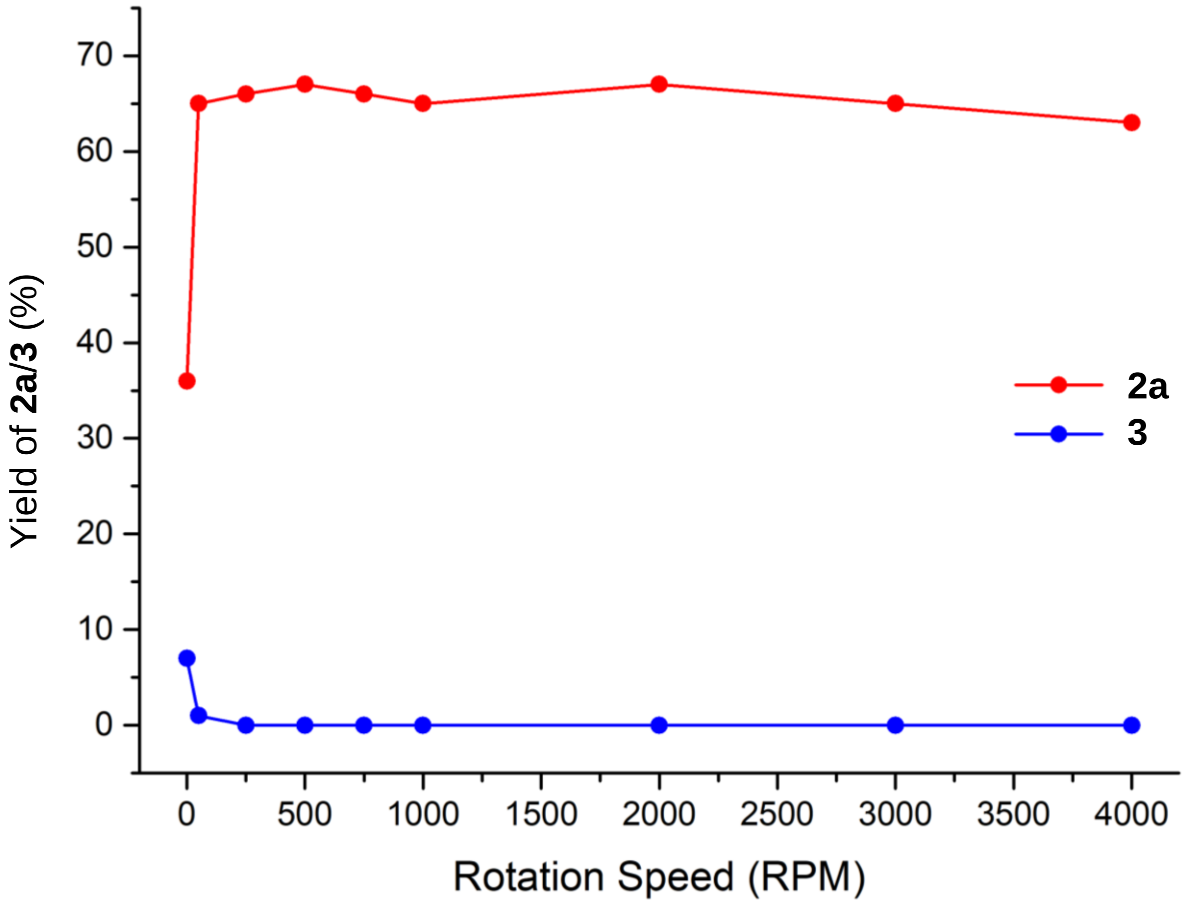

Figure S2. Study of rotation speed run at 520 mA and 2 mL min^{-1} flow rate. 0.14 M **1a** with 1.5 M LiBr and 6 equiv. DMU.

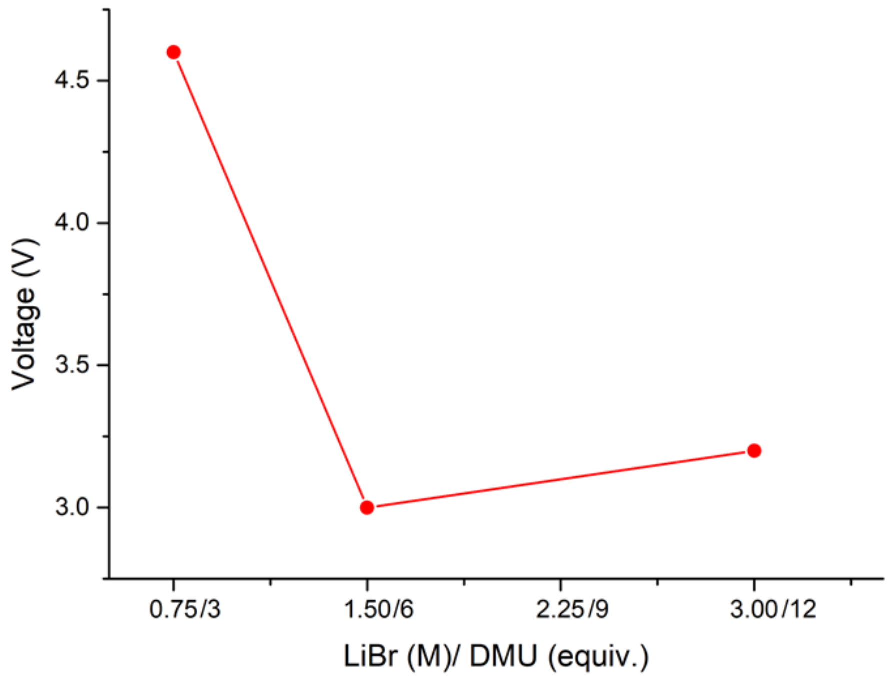

Figure S3. Showing voltage to maintain 520 mA for the different concentrations of LiBr and DMU at 1.75 mL min^{-1} flow rate.

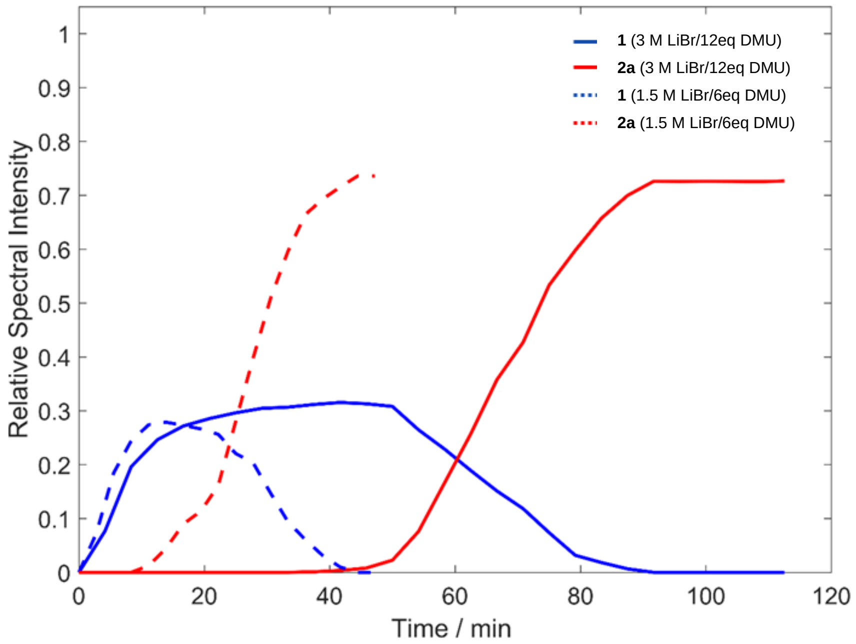

Figure S4. Showing the equilibration of the reactor as tracked by FTIR for the 3 M and 1.5 M solution at 520 mA and 1.75 mL min^{-1} flow rate.

Table S1. Preliminary Optimisation of conditions for the flow de-aromatisation of **1a** using **TPPA.**

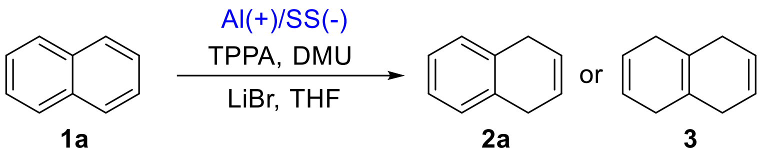

Entry	Flow Rate (mL min^{-1})	*I* (mA)	Conv. (%)	Selectivity of **2a** : **3**
1	1.75	800	100	100 : 0
2	1.75	800	39	11 : 28
3	1.75	400	100	72 : 28
4	1.75	200	100	67 : 33
5	3.50	1600	100	100 : 0
6	5.40	2400	100	94 : 6

Conditions: 4000 rpm, 0.14M **1a** with LiBr (7.5 equiv.), TPPA (10 equiv.) and DMU (3 equiv.) in THF. Conversion and product ratios determined by ^{1}H NMR analysis.

When the reaction mixture contained TPPA, in each case the reactor blocked between 20-30 min after the current was applied.

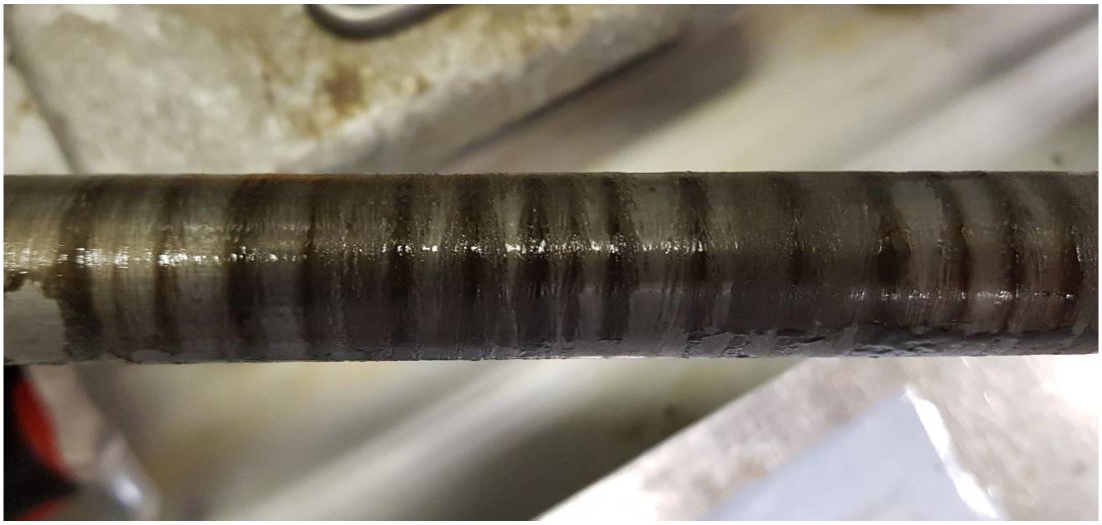

Figure S5. Showing the stainless steel rotor after a blockage occurred under the conditions using TPPA. The rotor was found to be coated in a black tar like material (this material also filled the annulus of the reactor).

Table S2. Preliminary Optimisation of conditions for the flow de-aromatisation of **1a.**

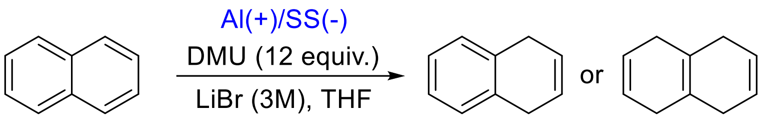

Entry	Flow Rate (mL min^{-1})	*I* (mA)	Deviations/ Additives	Conv. (%)[a]	Yield of **2a** (%)[a]	Yield of **3** (%)[a]
1	1.75	520	-	85	84	1
2	1.75	520	Sample taken after 3 reactor volumes	67	67	-
3	5	520	-	53	52	1
4	1	520	-	98	83	15
5[b]	1.75	520	TPPA (10 equiv.)	-	-	-
6	1.75	520*	*t*-BuOH (12 equiv.) as H^+ source	64	42	22
7	1.75	520*	NBu_4BF_4 (0.5 equiv.)	<1%	-	-
8	1.75	520	DMU (6 equiv.) LiBr (1.5M)	59	59	-
9	1.75	520	DMU (3 equiv.) LiBr (0.75M)	61	61	-

Standard conditions: 4000 RPM, 0.14M **1a**, 3M LiBr and 12 equivalents of DMU in THF. a) Conversion and yield determined by ^{1}H NMR analysis. b) Reactor blockage occurred. * = Current set but not achieved during the reaction.

Extended Reactor Operation

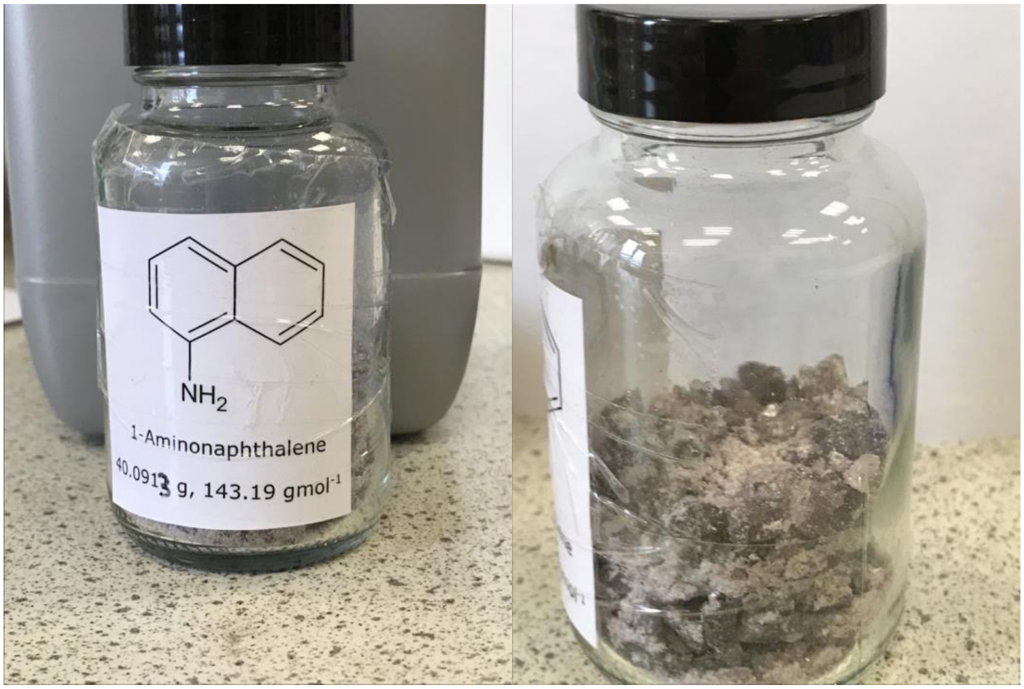

Figure S6. The substrate (**1b**) used for the extended run.

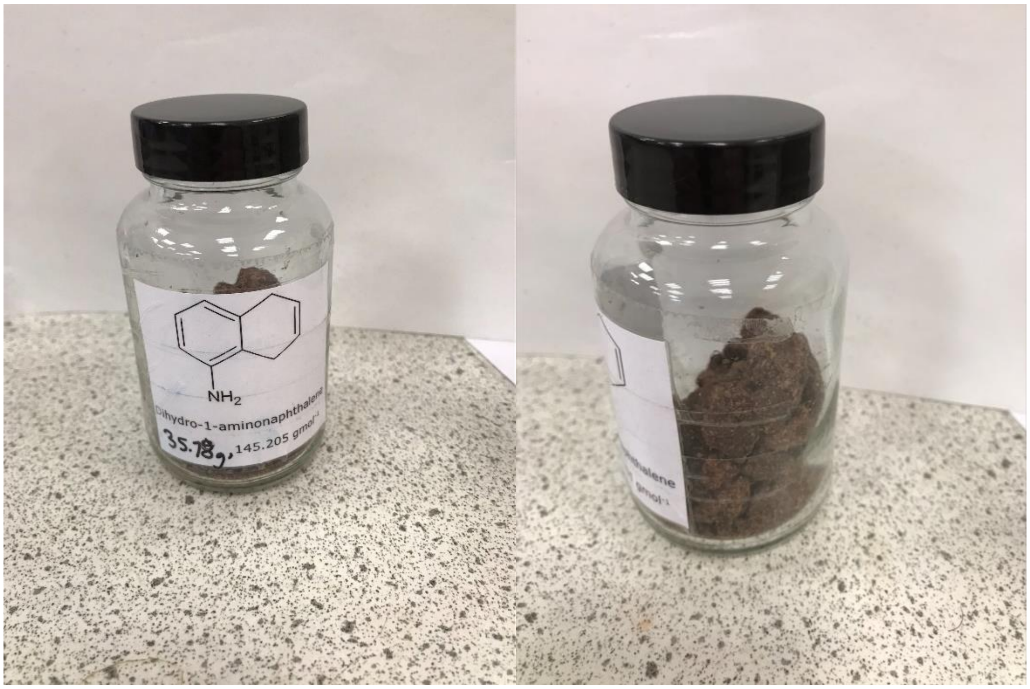

Figure S7. The crude product (**2b**) recovered from the extended 8hr run.

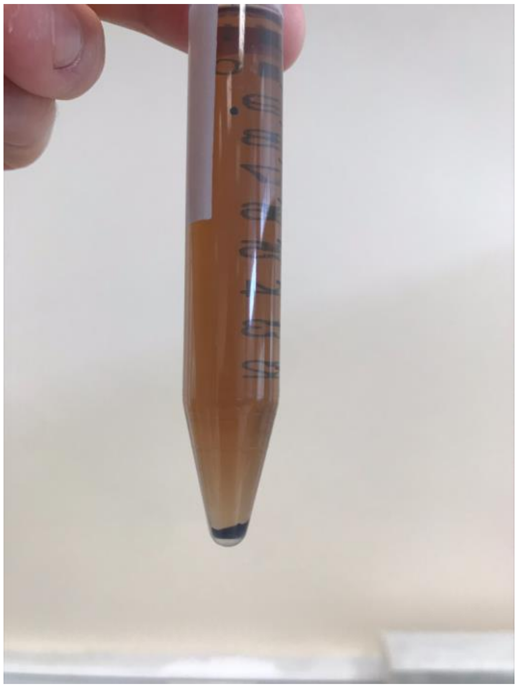

Figure S8. The reaction mixture after centrifuging to separate the black residue.

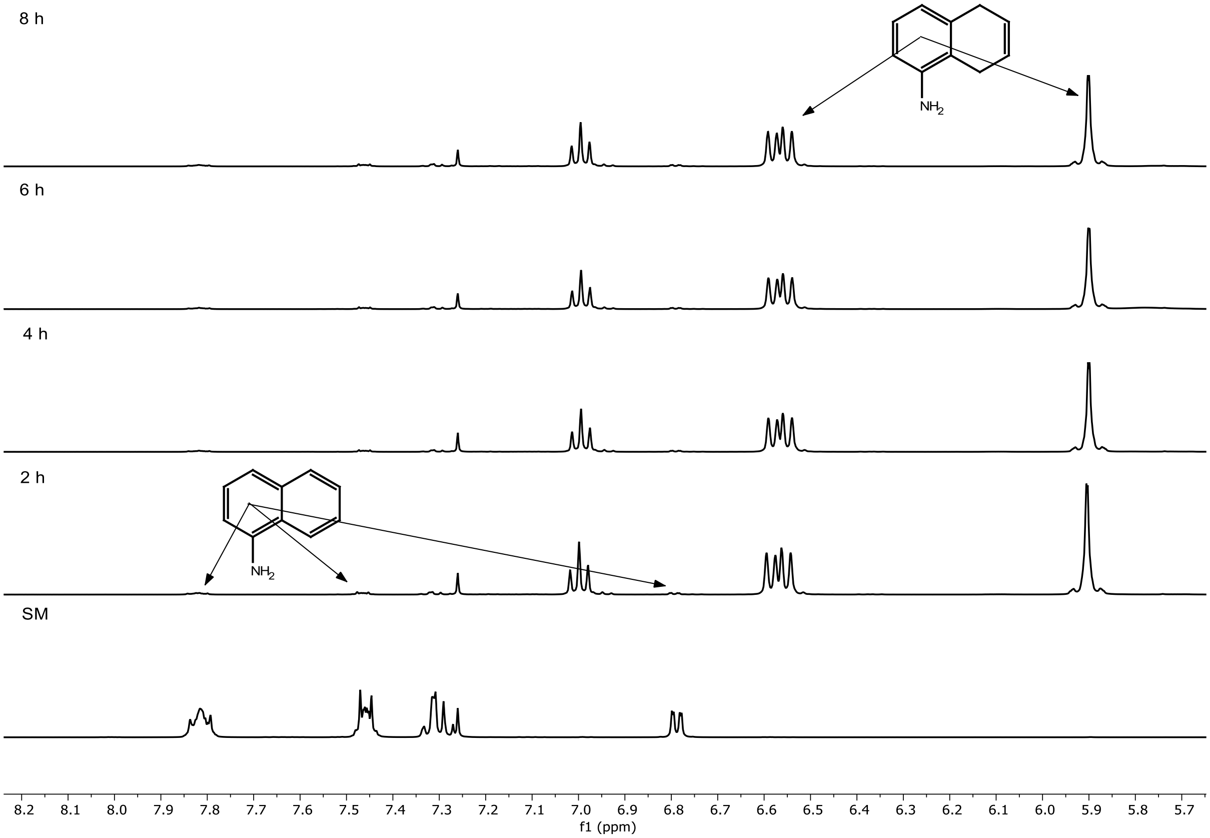

Figure S9. 1H NMR spectra of the crude reaction mixture (following mini work-up by extraction with 1.0 M aqueous potassium sodium tartrate), showing consistent >90% selectivty between the Birch reduction product and starting material.

Examination of the sacrificial electrode following the > 8 hr run.

The bore of the tube was measured at both ends prior to the experiment. At the end of the run, the electrode was cut in half at the point indicated by the blue bands. The bore was then measured at the red, green and two blue ends, Figure S10. The fluid flowed from the red end to green. Then the green/blue tubular section was cut in half longditudinally so that their interior surfaces could be examined (see Figure S11).

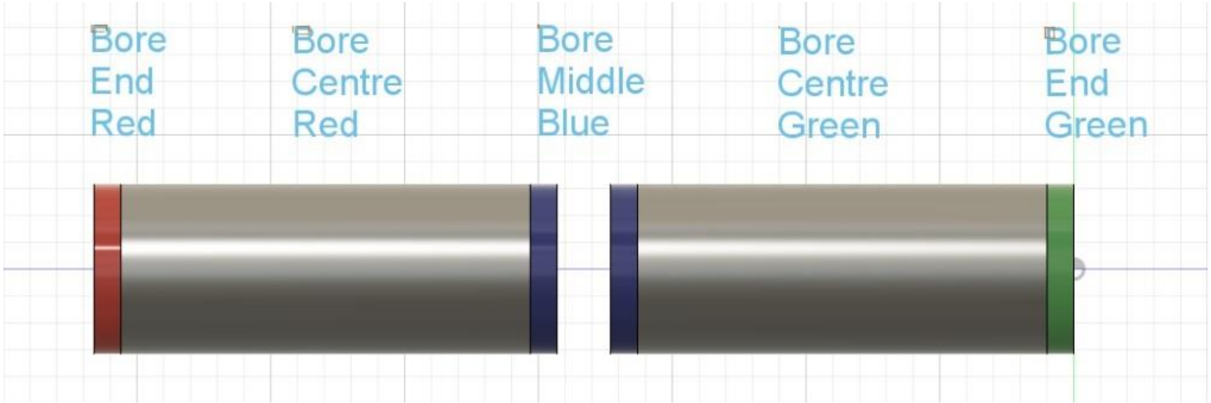

Figure S10. Diagram of the cut Al tubular electrode.

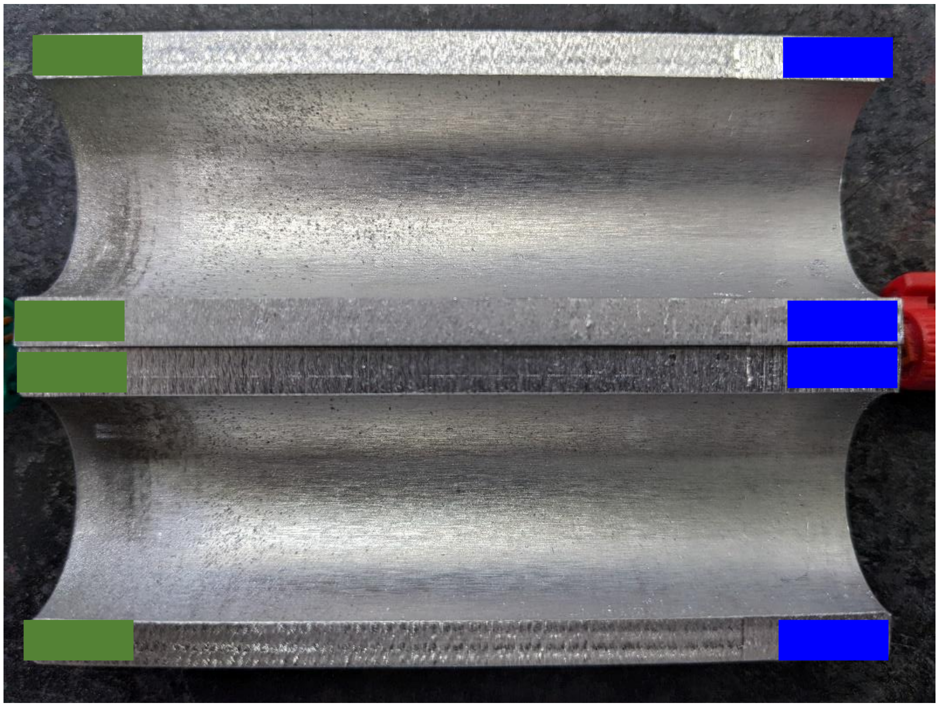

Figure S11. Cut Al tubular electrode. Note the traces of dark deposit at the green (outflow) end of the electrode. There is no evidence for any localised erosion of the surface. Visual inspection of the bore of the blue/red section did not reveal any obvious erosion – so it was not cut in half.

Table S3. Charaterisation of the outer Al tubular electrode after extended reactor operation

**Mass before)**	**Mass (After)**	**Bore End (Red) Bottom**	**Bore End (Green) Top**	**Bore Middle (Red/Blue)**	**Bore Middle (Green/Blue)**	**Bore Centre(Red)**	**Bore Centre (Green)**
(g)	(g)	(mm)	(mm)	(mm)	(mm)	(mm)	(mm)
192.4408	185.7080	22.425	22.295	22.39	22.4	22.415	22.4

Mass Loss = 192.4408 g – 185.7080 g = 6.7328 g = 0.25 mol Al

Average I.D after 8 h reaction = 22.388 mm

Additional FTIR Spectra

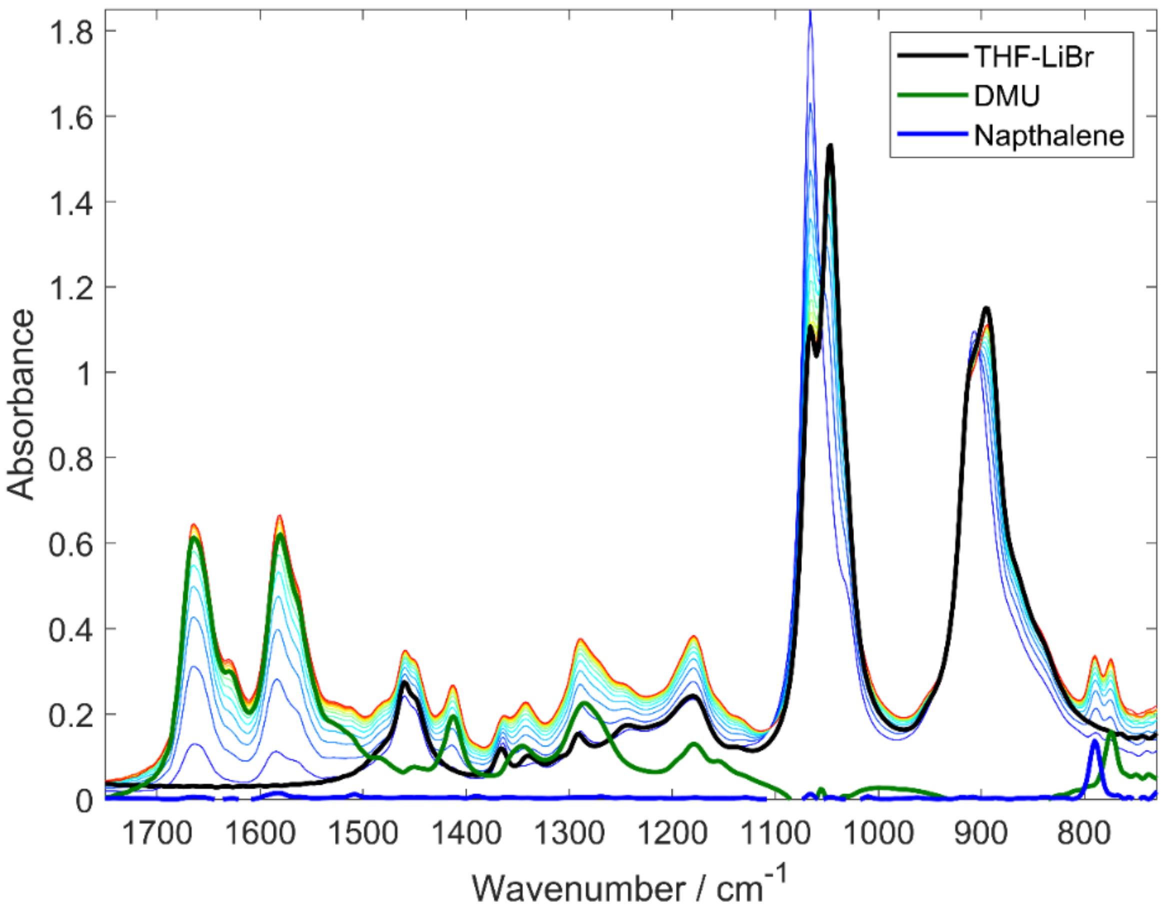

Figure S12. Infrared spectra collected as the reactor feed was switched from dry THF to the reaction mixture as it was introduced into the reactor and flowed out of the reactor outlet (Blue to Red, thin lines). Pure reference spectra of the individual mixture components are overlaid for comparison and characterisation (thick lines) This process was monitored via an ATR-FTIR fibre optic probe and allowed for the reactor dynamics to be monitored.

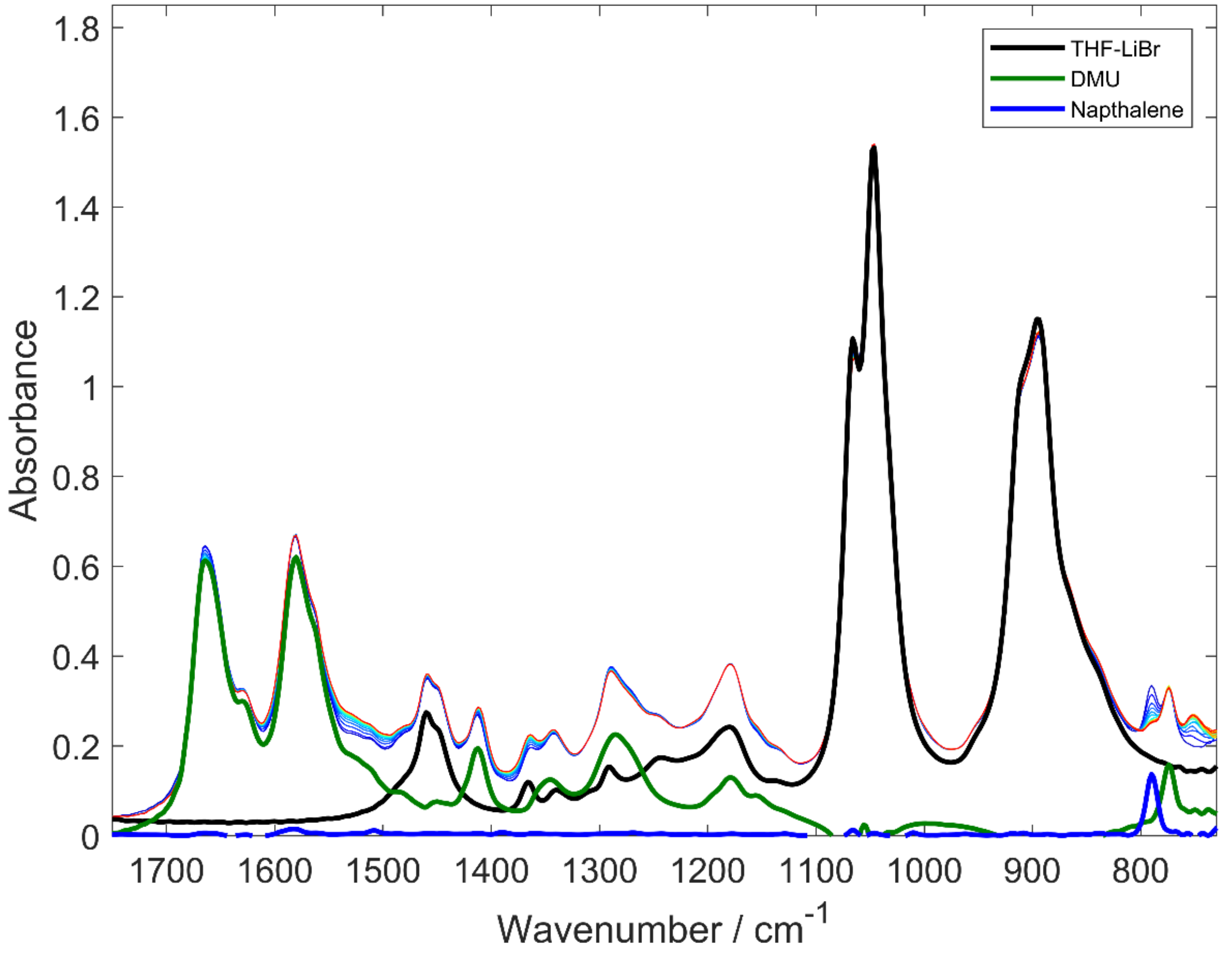

Figure S13. Infrared spectra of the reactor outlet (Blue to Red, thin lines) the current was applied to the reaction mixture and reduction began. A characteristic product band of the first reduction (1,4-dihydronaphthalene) can be observed to emerge at *ca.* 750 cm^{-1} as the primary naphthalene peak at *ca.*790 cm^{-1} decreases in intensity. Other changes can be observed in the spectrum between 1150-1700 cm^{-1} these relate to additional 1,4-dihydronaphthalene bands as well as a shift in the DMU peaks.

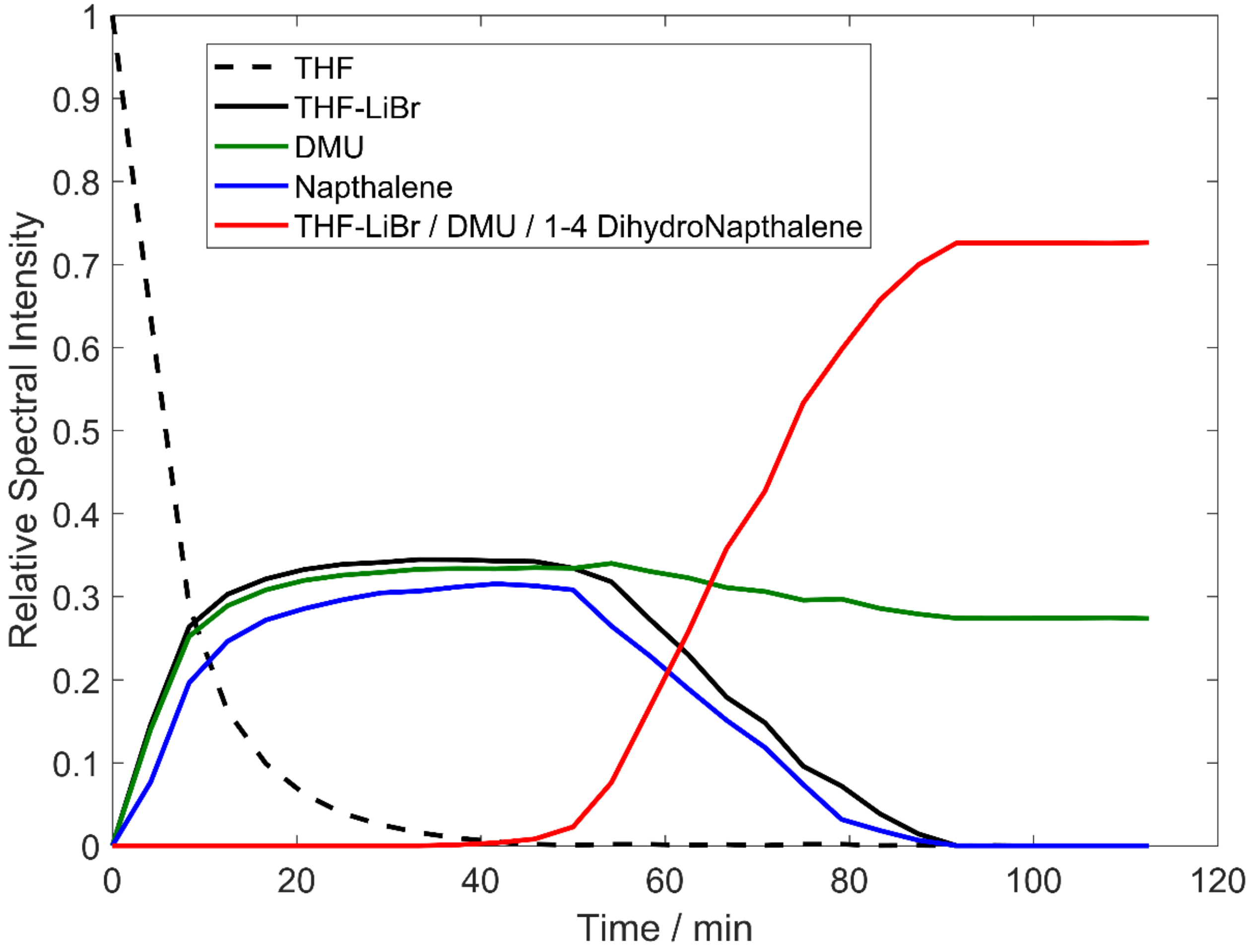

Figure S14. MCR could then extract the kinetics of this process from these factors relative spectral intensities within the infrared data, showing the dry THF leaving the system, being replaced by the starting mixture and then showing the conversion of the naphthalene to 1,4-dihydronaphthalene.

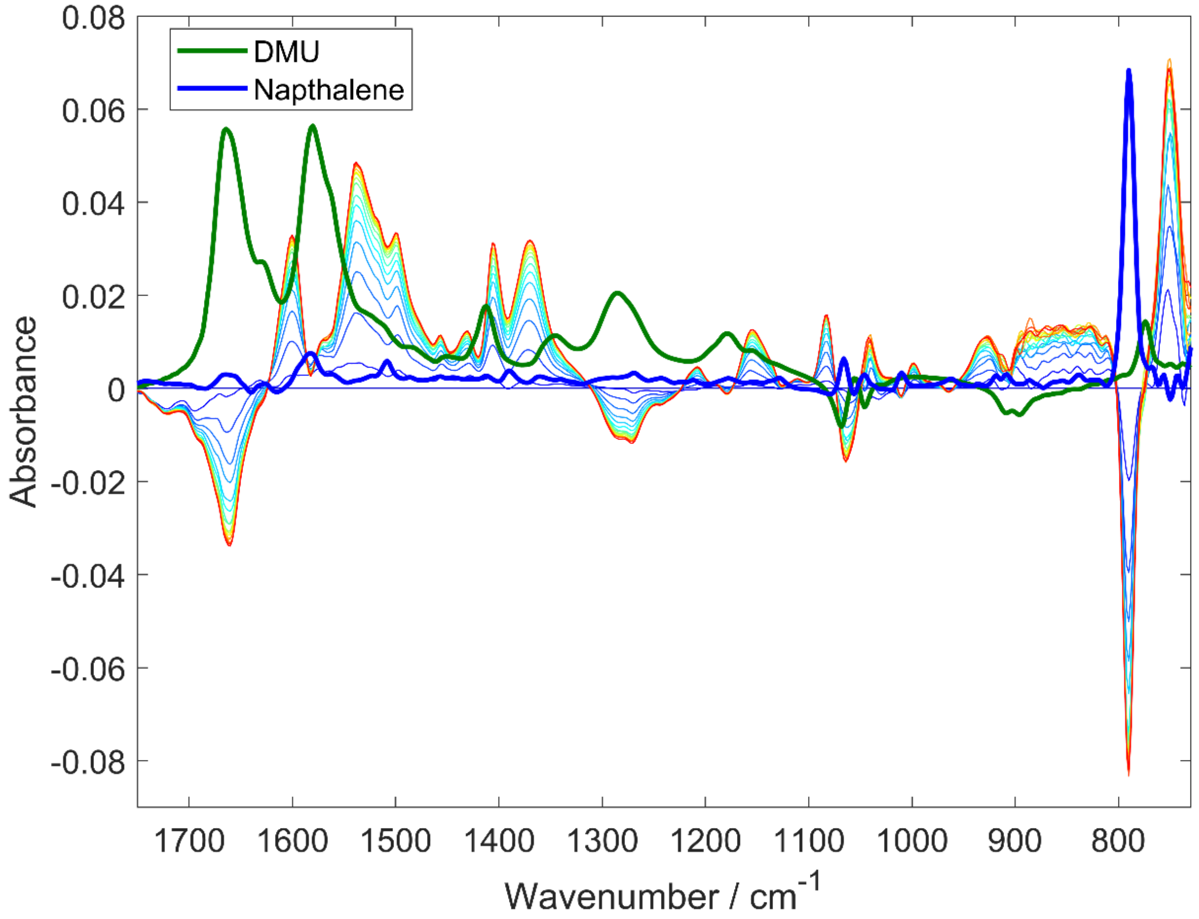

Figure S15. These changes can be more easily seen in this difference spectrum. This difference spectrum again shows the appearance product band at *ca.* 750 cm^{-1} and the disappearance of the naphthalene peak at *ca.* 790 cm^{-1}, as well the new product peaks at *ca.* 1200 cm^{-1} and shift in the DMU peaks. This shift in the DMU manifests as an increase in intensity around 1600 cm^{-1}, and a decrease around 1570 and 1670 cm^{-1}, resembling the difference observed by Baran and co-workers when DMU is removed from the presence of LiBr. This could suggest that the lithium is no longer complexing the DMU after the reduction has taken place.

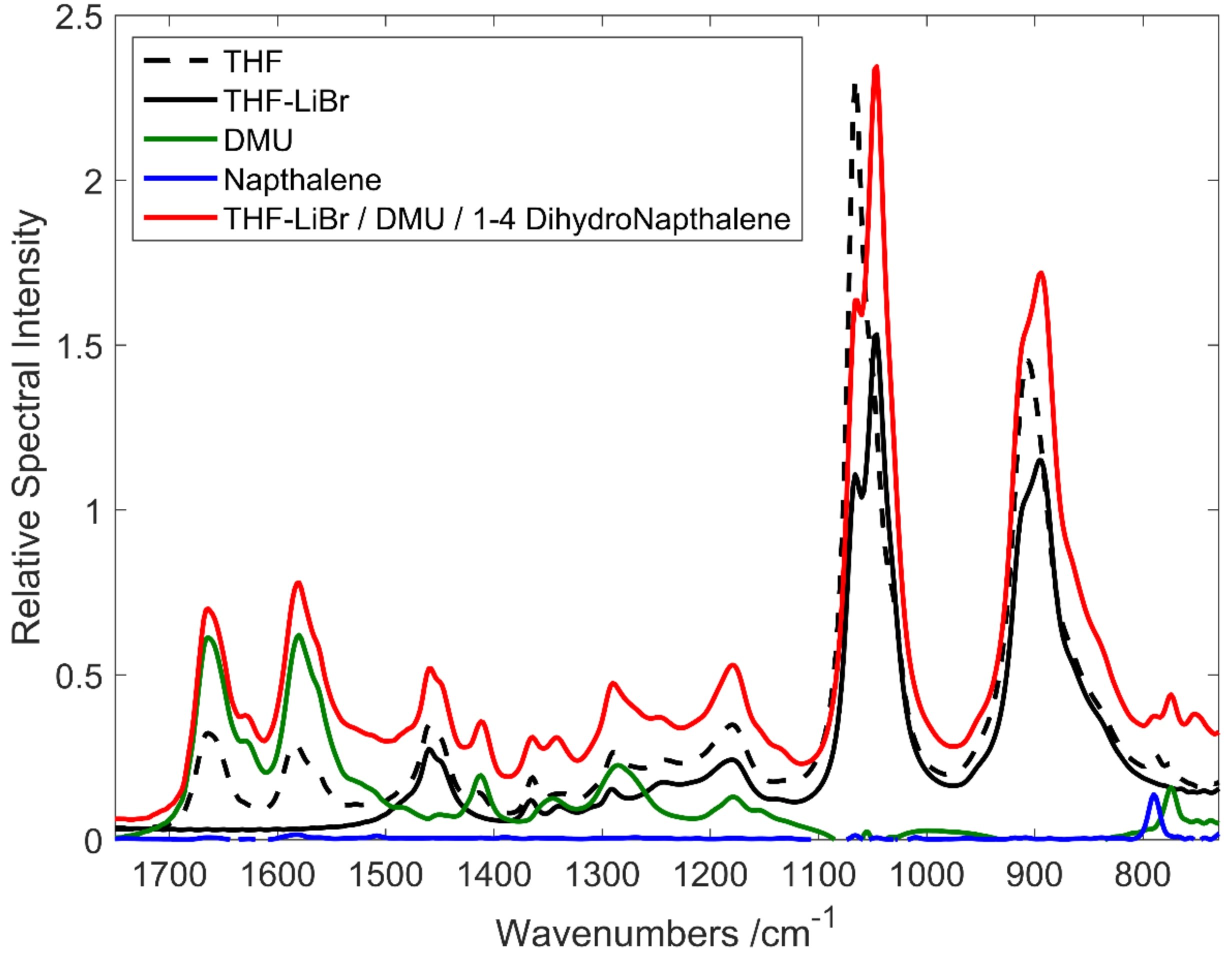

Figure S16 These data could then be analysed using multivariate curve resolution to extract he kinetic information from the process of introducing the reactants to the reactor, and then initiating the reduction. This was achieved by using the reference spectra as hard bounds in the algorithm, and then allowing for two extra "free" factors to be fit to the data in order to extract the rest of the constituents, (in this case the shifting THF-LiBr / DMU / 1-4 Dihydronaphthalene for product formation, and dry THF)

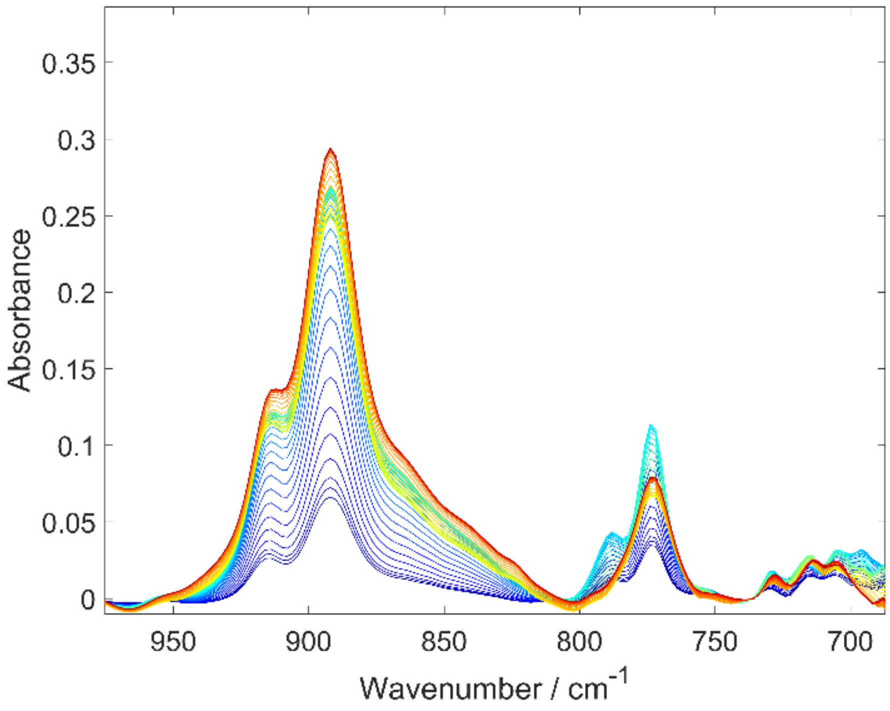

Figure S17. Inline FTIR spectra showing peaks relating to **1b** (~785 cm^{-1}) and DMU (~775 cm^{-1}) during the production of **2b,** demonstrating the need for Raman spectroscopy to probe <700 cm^{-1} in order to monitor the production of **2b** which possess characteristic vibrational modes in that range**.**

Additional Raman Spectra

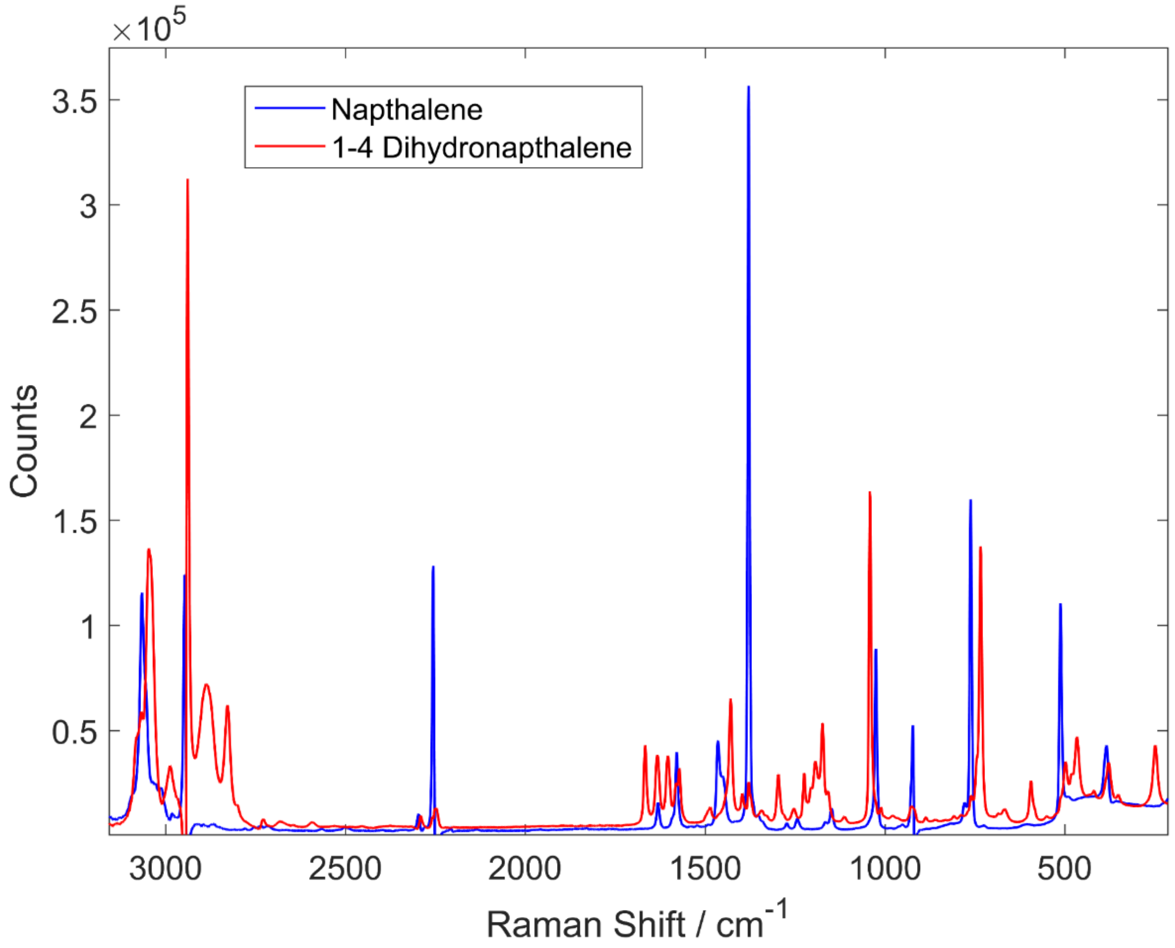

Figure S18. Raman spectra of components **1a** and **2a** with the solvent subtracted.

A-TEEM Spectroscopy

EEM spectra consist of an excitation emission matrix with an intensity axis, usually derived from number of photons of a given wavelength reaching the detector. This intensity is therefore given in counts can then be normalized between 0 and 1. This can be illustrated by the EEM spectrum of **2b**. as demonstrated in Figure S19.

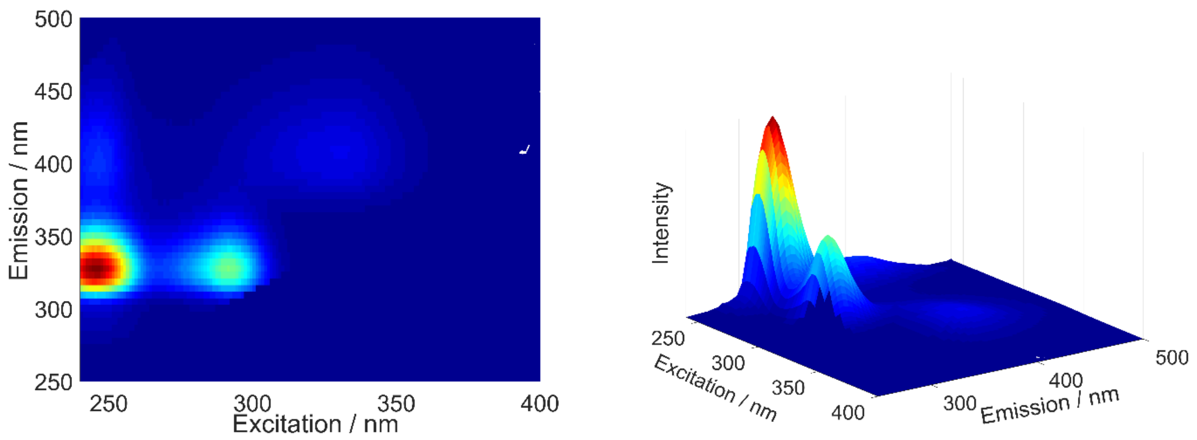

Figure S19 Normalized EEM Spectrum of **2b** as a contour plot (left) and 3D mesh (right). The normalized EEM spectra can be depicted as a contour plot of excitation vs. emission vs. fluorescence intensity (Blue: low intensity, Red: high intensity) or as a 3D projection, In this paper, we have used the 2-D projections for simplicity.

Computation Fluid Dynamic (CFD) Details

The CFD simulations were conducted using the commercial software ANSYS-Fluent 2022R1. The modelled electro-vortex reactor was two dimensional and axisymmetric with a mesh size of 146k nodes. The CFD method is validated with the experiemtnal data from literature (*J. Colloid Interface Sci*. **2005**, 285, 167-178), where the velocity profiles are used for the comparison. A species of a mass fraction of 0.1 that has the same properties of the working solution is injected from the rotor to model the electro-reaction. At low rotation speed (e.g., 100 RPM), the flow in the reactor is laminar, but at high rotation speed (e.g., 4000 RPM), the flow is turbulent and was modelled using the k-w turbulence model. The laminar case was modelled without acceleration due to the low rotation speed, thus, the simulation was initiated from the rest. However, for the turbulent case, an acceleration of 500 RPM is used progressively starting from the laminar case (e.g., 100 RPM) till the desired rotation speed 4000 RPM is reached. The transition from laminar to turbulent regime is estimated based on the Reynolds number ratio (actual rotation Re_{Ω}/critical Re_{cr}). The Re_c is equal to 109 for the present reactor radius ratio of η=Ri/Ro=0.8636. Thus, if the Re_{cr} ratio > 20 the flow is turbulent and if its < 20 the flow is laminar. The parameters of the simulations are:

Rotor radius (Ri) = 9.5 mm

Vessel radius (Ro) = 11 mm

Gap size = 1.5 mm

Solution 1 (0.75 M): dynamic viscosity = 0.00069 kg/m s; Re ratio (4000 rpm) = 76.11

Solution 2 (1.5 M): dynamic viscosity = 0.00092 kg/m s; Re ratio (4000 rpm) = 59.16

Solution 3 (3 M): dynamic viscosity = 0.00253 kg/m s; Re ratio (4000 rpm) = 24.325

Flow rate = 1.75 mL/min

Rotation speed = 100, 200, and 4000 rpm

The mixing efficiency is calculated using the following formula:

$$M_{eff} = \left(1 - \frac{Y_{i,ini} - Y_{i,area-ave}}{Y_{i,ini}}\right) \times 100$$

where $Y_{i,ini}$ is the initial mass fraction injected from the rotor and equal 0.1, and $Y_{i,area\text{-}ave}$ is the area-averaged mass fraction of the injected species through the whole reactor.

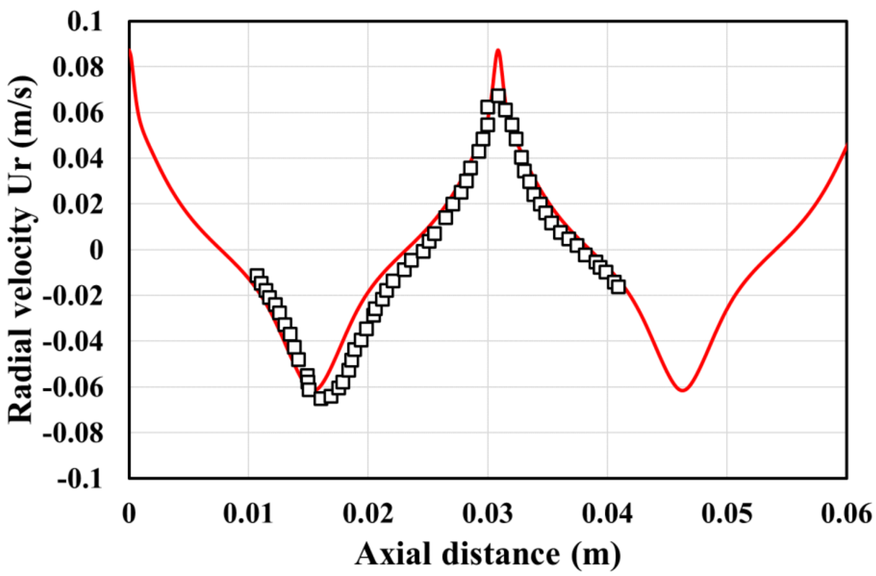

Figure S20. Validation of the present CFD modeeling with the experimental results from the literature (*J. Colloid Interface Sci*. **2005**, 285, 167-178). Profiles showing the radial velocity through Taylor vortices for a turbulent Reynolds number ratio of 34.

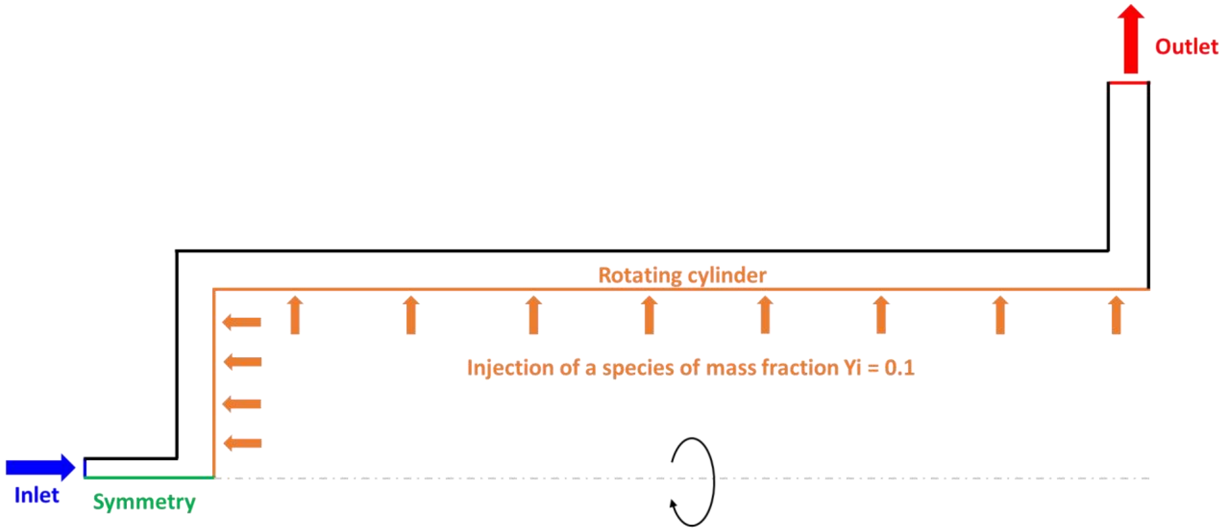

Figure S21. The modelled 2D symmetric domain. At the inlet, a mass flow rate in kg/s is imposed, which varies from one solution to another due to the viscosity change. All walls (the rotor and vessel) are assumed adiabatic with non-slip condition. A species of a mass fraction of 0.1 is injected through the rotor. At the outlet a static pressure condition is used.

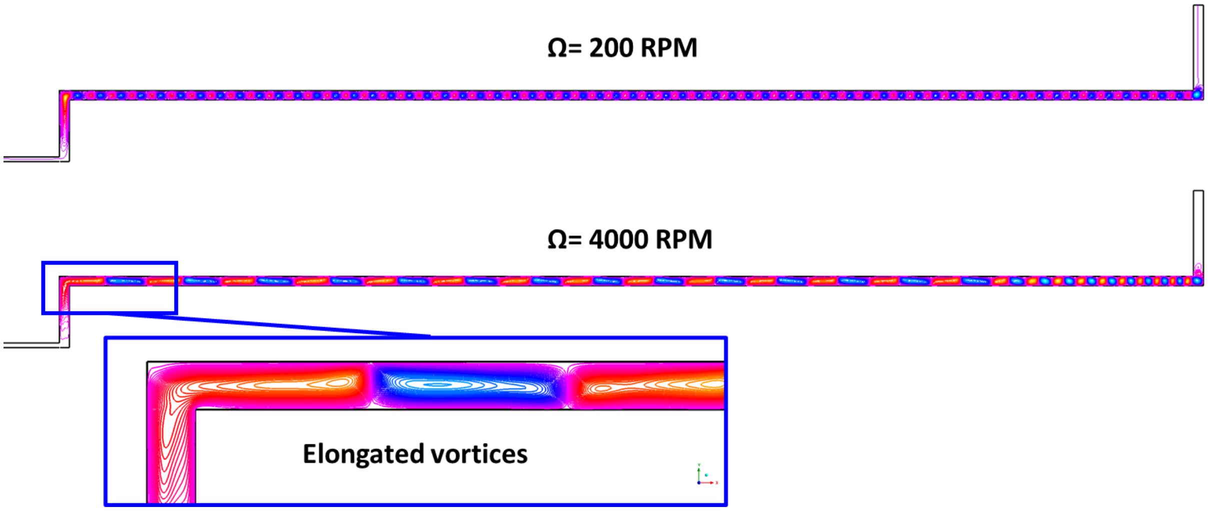

Figure S22. Showing the flow field of the 3M solution inside the reactor at 200 rpm (top) and 4000 rpm (bottom). Taylor vortices visualised using streamlines are found to be elongated at high rotation speed (4000 rpm) due to the high momentum transferred from the rotor to the solution. The elongated vortices lead to increase mixing within the gap.

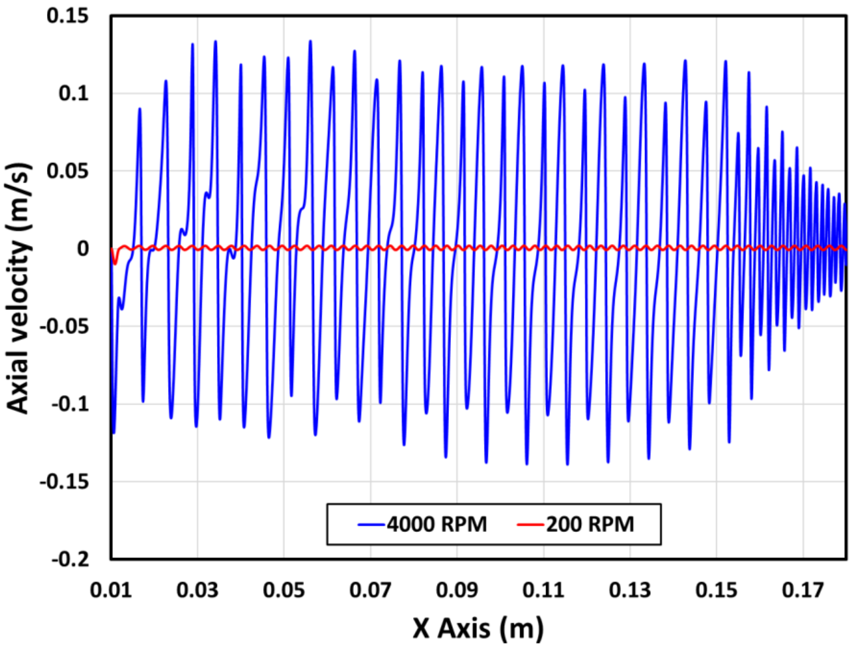

Figure S23. Showing plots of the axial velocity of the 3 M solution at the mid gap through the whole reactor annulus. High rotation speed (4000 rpm) generates higher rotation momentum within the vortices and consequently high axial velocity, which is 7 times order of magnitude of the 200 rpm axial velocity case. Non-elongated vortices at the end of the reactor (~X>0.15m) at 4000 rpm show low rotation momentum.
